# Helix kinks are equally prevalent in soluble and membrane proteins

**DOI:** 10.1002/prot.24550

**Published:** 2014-04-16

**Authors:** Henry R Wilman, Jiye Shi, Charlotte M Deane

**Affiliations:** Department of Statistics, University of Oxford1 South Parks Road, Oxford, OX1 3TG, United Kingdom; UCB Celltech, Branch of UCB Pharma S.A.208 Bath Road, Slough, SL1 3WE, United Kingdom

**Keywords:** membrane protein, protein structure, protein helix, helix kink, helix distortion, soluble protein, helix bend

## Abstract

Helix kinks are a common feature of α-helical membrane proteins, but are thought to be rare in soluble proteins. In this study we find that kinks are a feature of long α-helices in both soluble and membrane proteins, rather than just transmembrane α-helices. The apparent rarity of kinks in soluble proteins is due to the relative infrequency of long helices (≥20 residues) in these proteins. We compare length-matched sets of soluble and membrane helices, and find that the frequency of kinks, the role of Proline, the patterns of other amino acid around kinks (allowing for the expected differences in amino acid distributions between the two types of protein), and the effects of hydrogen bonds are the same for the two types of helices. In both types of protein, helices that contain Proline in the second and subsequent turns are very frequently kinked. However, there are a sizeable proportion of kinked helices that do not contain a Proline in either their sequence or sequence homolog. Moreover, we observe that in soluble proteins, kinked helices have a structural preference in that they typically point into the solvent.

## INTRODUCTION

Disruptions of α-helices (frequently referred to as kinks) are known to occur in many proteins.^1^–^3^ It has been claimed that these are much less frequent in soluble protein helices than membrane protein helices,^4^–^6^ and that those in transmembrane helices are linked to function.^7^–^15^ The majority of previous research has concentrated on kinks in transmembrane helices, but there is little comparable research into soluble helix kinks.

In the realm of transmembrane helices, there is disagreement about the definition, identification, and causes of helix kinks. Several studies have tried to annotate kinks, each using a different method (e.g., Refs.^6^, ^16^–^21^). While some methods have a binary classification of helices (kinked/straight^16^,^18^,^19^), others have a ternary system (kinked/curved/straight^17^,^20^ or kinked/distorted/straight^6^). The algorithms used to define the helix set also differ, these studies use methods such as DSSP,^18^,^22^ the PDB annotation,^19^,^23^ or manual inspection.^17^ As a consequence, the methods do not consistently identify the same set of helices as kinked.

The number of kinked helices identified differs from study to study; ranging from 6% kinked (although a further 59% of helices are annotated as curved),^20^ through 44%,^19^ 45% (19% curved),^17^ and 60%,^16^ to 64% kinked.^6^ After kink annotation in transmembrane helices it is then possible to investigate residue types around a kink (e.g., the presence of Proline), or other indicators of kinks (e.g., absence of a backbone hydrogen bond). Such characteristics can be used to predict which helices are kinked.^16^–^18^

Hydrogen bonds between the amide group of the *i* + 4th residue and the carbonyl group of the ith residue in the backbone of the protein are the primary feature of α-helices. A missing *i* + 4 → *i* hydrogen bond is frequently observed close to the kink in a kinked helix. Kinks may be stabilized by the presence of non-canonical hydrogen bonds, such as *i* + 3 → *i* or *i* + 5 → *i* backbone connections, sidechain to backbone,^19^,^24^ or other types of hydrogen bonds.^25^–^27^ These non-canonical hydrogen bonds have also been implicated in the flexibility of membrane helix kinks, by residues shifting their backbone hydrogen bond partners.^4^

The presence of Proline in a helix is strongly associated with that helix being kinked.^3^,^6^,^12^ Proline cannot be fully incorporated into an α-helix, due to its lack of an amide proton, and the ring formed by its backbone and sidechain.^19^,^28^,^29^ The lack of an amide proton prevents the canonical *i* + 4 → *i* backbone hydrogen bond, and the ring is not easily accommodated in the helix structure. Although earlier work suggested that all kinks were associated with Prolines, or so-called vestigial Prolines (where Proline is not seen in the kinked helix structure, but is observed in a homologous sequence),^12^ more recent studies have shown that there are many kinks that are not associated with Proline.^4^,^19^

In addition to Proline, studies have suggested other amino acids that could be important in kink occurrence, but none have been consistently identified. For example, Glycine has been found to be prevalent around kinks in some studies,^5^,^17^,^19^ but not in others.^6^,^16^,^18^ Similarly, Serine has been identified as a factor in kinks in some studies,^17^,^19^,^30^ but not in all.^6^,^16^,^18^ It has also been suggested that residues with sidechains that can form hydrogen bonds to the backbone, such as Serine and Threonine, are important in kinks,^8^,^24^ although these side chains are rarely seen bonding to the backbone in the known structures of kinks.^19^,^24^ A number of articles have suggested more complex sequence motifs that may be important in kinks (e.g., Refs.^19^, ^30^–^32^), but once again none of these motifs are consistently observed across studies. These studies have a similar approach to their choice of data set. They are compiled from the PDB,^23^ and use one of the membrane protein databases (e.g., PDBTM,^33^ MPtopo,^34^ or OPM^35^) to identify membrane helices.

Although the amount of membrane protein structure data is ever increasing over time, the size of the data set also depends on the chosen redundancy and quality thresholds. The number of helices used varies in the different studies, from 405 in Hall *et al*.,^19^ to 1014 in the work of Kneissl *et al*.^17^ In this study, we use 1208 helices taken from 268 protein chains.

This highlights one of the difficulties with the above body of research. It focuses on membrane helices and is therefore hampered by the small amount of membrane structure data available. If the membrane protein structures available in 2011 were made non-redundant to 80% sequence identity, the remaining set would have contained only 1014 helices from 132 protein chains,^17^ within which there would be 300–500 kinks. A higher redundancy threshold gives a larger data set, which allows stronger statistical conclusions to be drawn. However, increasing the redundancy raises the chances of bias.

The differences in the results of previous studies may also be due to the different methods used to identify kinks, and specifically the different way in which each study identifies the kink residue. In this study, we use a method to consistently identify the kink residue with respect to the geometry of the kink.

There are far more solved structures of soluble proteins, than there are of membrane proteins, with much higher diversity. Taking advantage of this data we compare helix kinks in soluble and membrane proteins. Our aim is to identify the similarities and differences of kinks in membrane and soluble helices, to determine what patterns of soluble helix kinks can be applied to kinks in membrane protein helices.

Kinks in soluble proteins have not been studied to the same degree as those in membrane proteins. There are examples of functional kinks in soluble-protein helices,^36^,^37^ and we have found two examples of research on general analysis of helix distortions in soluble proteins. In one analysis of soluble Helix-X-Helix motifs, kinks were usually found when the linker residue (X) was Glycine, Serine, Aspartic acid or Asparagine, or when Proline was found after the linker residue.^5^ Additionally, the linker residue was frequently buried. A study of Proline distortions in soluble protein helices focused on classifying the distortions based on Ramachandran angles and hydrogen bonding patterns, however, unlike the studies of membrane helices, it did not consider the residue preferences around these distortions.^6^,^17^–^19^

Although kinks are thought to be more frequent in membrane protein helices than soluble protein helices,^4^–^6^ to our knowledge there is no study that makes a direct comparison between the frequency and features of kinks in helices of membrane and soluble helices. In this article we investigate kinks in both membrane and soluble protein α-helices. We demonstrate that kinks are equally prevalent in the two types of proteins. Specifically we find that kinks occur in long helices, and kinks occur equally frequently in long helices regardless of their environment. Kinks in the two types of proteins are similar in their residue patterns, and frequency of broken hydrogen bonds. Proline is a dominant feature of kinks in both types of protein, while other residue patterns are similar, allowing for the expected differences in the amino acid distributions. One notable feature that occurs only in soluble protein helices is that kinks have a structural preference to point into the solvent, revealed by patterns in both the hydrophobicity and solvent accessible surface area of residues around the kink.

## MATERIALS AND METHODS

Two sets of protein helix structures were collected from the PDB,^23^ one containing soluble protein helices, and the other transmembrane helices.

### Soluble data set

For soluble proteins, the PDB was filtered with PISCES,^38^ using the following settings: 80% sequence identity, 40 < chain length < 1000 residues, resolution <5 Å, R-factor <0.4, include non X-ray structures, exclude Cα-only structures. We use the first conformer in each NMR protein structure. To remove any transmembrane structures from this set, any protein chains that were in the PDBTM,^33^,^39^ and/or in the Membrane Proteins of Known Structure Database^40^ were excluded. The JOY program was used to annotate the helix structures.^41^ Initially, regions of contiguous helix were identified, using the DSSP algorithm.^22^ Those helical regions separated by only one or two coil residues were combined. These helices were split if they contained a kink angle, as defined by Kink Finder (this work; see Methods section), >60°.

The ends of the helices were checked for helical nature. Where the first five and last five residues of the helix were not a helical seed, as defined in MC-Helan,^6^ residues were iteratively removed from the end of the helix, until this condition was met. The requirements for a helical seed are threefold. First, the first residue must have dihedral angles in the alpha-helical region.^42^ Second, the angles (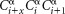) must lie within the expected ranges for an alpha helix (35°–50° for *x* = 2, 60°–80° for *x* = 3, and 45°–65° for *x* = 4). Third, the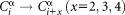 distances must be within 0.5 Å of the values for an ideal α-helix. Helices with 12 or fewer residues were removed, as this is the shortest length for which kinks can be identified by Kink Finder. The final data set contained 9742 protein chains, with a total of 29,699 helices. We also compiled data sets with three other sets of thresholds. Using 80% sequence identity, resolution <5 Å, R-factor <0.9, gave a set with 31,633 helices from 10,822 chains. Using 80% sequence identity, resolution <3 Å, R-factor <0.9, gave a set with 29,064 helices from 9528 chains. Using 50% sequence identity, resolution <5 Å, R-factor <0.9, gave a set with 24,245 helices from 7992 chains. These sets gave very similar results to each other (see Supporting Information Figs. S9–S11).

### Membrane data set

PDB codes of membrane proteins were initially identified from the Membrane Proteins of Known Structure Database^40^ and from the PDBTM. Structures derived from electron microscopy experiments, and those containing only Cα atom coordinates were removed. These proteins were split into their constituent chains, and filtered using the same criteria as for the soluble data set. The membrane position for each residue was annotated by iMembrane,^43^ with only chains annotated with ≥1 residue in the tail region of the membrane retained. All remaining non membrane proteins were removed by visual inspection. Only 20 chains had resolution ≥4 Å. Helices were identified in the same manner as for the soluble data set. Helices that had no residues annotated as being located in the tail region of the membrane by iMembrane were removed. In this way, the membrane annotation provided by iMembrane allows us to exclude non-membrane helices, such as the so called H8 helix in GPCRs, from this dataset. Helices were trimmed so that no more than five residues at either end were outside the membrane. This allowed Kink Finder to calculate an angle for each of the residues in the head or tail region of the membrane. The final data set contained 268 protein chains, with a total of 1208 helices (see Table S5 in the Supporting Information). We also compiled data sets with three other sets of thresholds. Using 80% sequence identity, resolution <5 Å, R-factor <0.9, gave a set with 1326 helices from 281 chains. Using 80% sequence identity, resolution <3 Å, R-factor <0.9, gave a set with 861 helices from 196 chains. Using 50% sequence identity, resolution <5 Å, R-factor <0.9, gave a set with 1002 helices from 220 chains. These sets gave very similar results to each other (see Supporting Information Figs. S9–S11).

### Length matching

Sets of soluble helices with the same length distribution as the membrane helices were selected by sampling from the full soluble helix set. For each sample, soluble helices are chosen, without replacement, to match the length distribution of the membrane helices. This is possible as currently there are far more structures of soluble helices than membrane helices.

### Sequence homolog collection

Homologous sequences for each protein chain in the two sets were collected. Sequences were obtained by using PSI-BLAST^44^ to search the UniProt90 database,^45^ with the following settings: 2 iterations,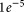 as the E value threshold (to include a sequence in the model used by PSI-BLAST to create the Position Specific Substitution Matrix, or “inclusion E thresh”), and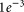 as the E value threshold (to retain a sequence). Sequences with a large length difference to the structure were removed (those with length >3/2 or <2/3 of the structure). The remaining sequences were aligned using MAFFT 7.015.^46^ Protein sequence profiles were built using the sequence alignments, with each sequence weighted according to its similarity to every other sequence, with more dissimilar sequences having higher weights.^47^–^49^ Helices had on average 52 homologous sequences. The homologous sequences were used only in the calculation of amino acid propensities and hydrophobicities.

### Hydrophobicity

Amino acid hydrophobicities are taken from experimentally derived interface-octanol data.^50^ The sequence profiles derived from sequence homologs were used to calculate the hydrophobicity of each position near the kink. In the profiles, each sequence was weighted according to its similarity to every other sequence, with dissimilar sequences given higher weights.^47^–^49^

### Hydrogen bonds and solvent accessible surface area

The JOY program was used to annotate both backbone and sidechain hydrogen bonds in the protein structures, and the solvent accessible surface area of each residue.

### Kink identification

Kinks in helices were identified by the Kink Finder algorithm. An outline of the method is shown in the Supporting Information (Fig. S1). Kink Finder calculates the local axis of the helix by fitting a cylinder to the backbone atoms of a sliding window of six residues. This window slides one residue at a time. Local kink angles (one per residue, except at the ends of the helix) are calculated from the angle between consecutive cylinder axes.

Kinks are identified at the residue with the largest angle in each helix, if it is over 20°. Further kinks are identified if the next largest angle is ≥20°, at least four residues from the first kink, and there is a residue with an angle ≤10° between the residue and the first kink. The residue identified as the site of the kink is chosen from the residue with the largest angle, the one before and the two after. The kink residue is the one of these with its wobble angle^51^ closest to 0° (for an outline of the method, see Fig. S2 in the Supporting Information). The wobble angle for a residue is calculated from the position of its Cα atom relative to the local helix axes. Two vectors are calculated. First, the C-terminal local axis is projected onto a plane perpendicular to the N-terminal local axis and containing the Cα atom. Second, the vector which is perpendicular to the N-terminal axis, and passes through the Cα atom. The “wobble angle” is the angle between these two vectors (see Fig. S3 in the Supporting Information). By selecting the kink residue to be on the inside of the kink based on the wobble angle we are effectively geometrically aligning the kinks.

For comparison, identical analysis was undertaken using kinks identified by the MC-Helan^6^ algorithm. By applying an angle cut off that classified a similar proportion of helices as kinked, and modifying the algorithm to select the kink residue by the same method as Kink Finder (as in Fig. S2 in the Supporting Information), we obtained results that had good agreement with those from the Kink Finder analysis. These results are shown in the Supporting Information Figures S4, S5, S8, and Table S1.

### Propensities

Amino acid sequence profiles can be used to examine whether residues are favored at specific positions around a kink, using their propensity to be at a given position relative to a kink, as shown in Eq.: 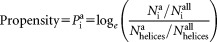
1where *N* is the number of residues, *N*_i_ is the number of residues at a given position, i, relative to the kink residue.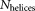 is the total number of residues in the helices in the data set being analyzed,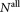 is the total number of residues, and *N*^a^ is the number of residues of a particular type (a), for example, glycine. The background distribution (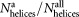) comes from the set of helices from which the kinks are identified. The data used to calculate the propensities come from the sequence profiles, which are compiled using homologous sequences. Protein sequence profiles were built using the sequence alignments, with each sequence weighted according to its similarity to every other sequence, with more dissimilar sequences having higher weights.^47^–^49^

### Motifs

Sequence motifs were identified by searching 13 residue segments around each kink, and the proportion of kinks containing one or more instances of the motif was recorded. This value was compared with a background, calculated by selecting a random 13 residue segment from each of the straight helices (those with maximum angle ≤14°) in the set, and searching these for the motif. This random sampling was repeated 50 times, and the result averaged.

The motif notation is based on regular expressions. Each character represents a single amino acid, the single letter amino acid identifiers are used, *x* represents any amino acid, and letters in square brackets (e.g., [ACD]) represent any one of the letters in the square brackets (e.g., A, C, or D). The “G_0_ or G_+1_” motif is a special case, which represents all segments where Glycine is at position 0 or +1.

## RESULTS

### Angle distributions

**Figure 1:**
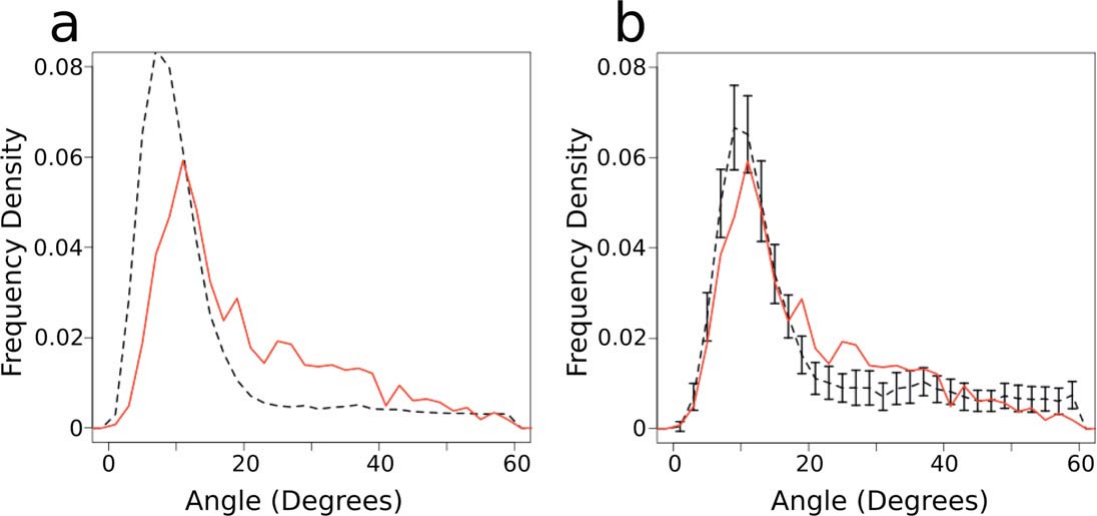
Kink angle distributions. (a) The distribution of maximum angles in all membrane (*solid red*) and all soluble (*dashed black*) helices. (b) The distribution of maximum angles in length matched sets of membrane and soluble helices. The soluble values are means of 50 samples which are matched to the same length distribution as the membrane helices. Again, the solid red line shows the membrane distribution, while the dashed black line shows the soluble distribution. Bars show 2 s.d. from 50 samples. Frequency density is the normalized frequency of helix length, such that the area beneath the graph is equal to one. [Color figure can be viewed in the online issue, which is available at wileyonlinelibrary.com.]

A simple comparison of the largest angle of each helix suggests that the proportion of highly kinked helices is lower in soluble proteins than in membrane proteins [Fig. 1(a)]. However, the distribution of the lengths of the two types of helices are markedly different, and the maximum kink angle in the helix is dependent on the length of the helix (see Figs. S6, S7, and S8 in the Supporting Information). We compared the maximum angle distributions of soluble and membrane helices of the same length (e.g., all 20 residue long soluble helices compared with all 20 residue long membrane helices), using a two sample Kolmogorov–Smirnov test.^52^,^53^ For the majority of lengths, the distribution of angles for membrane and soluble helices are not different (*P* value ≥0.05, *P* values for each test are shown in Table S2 in the Supporting Information). For shorter helices (lengths of 19 residues and shorter), the Kolmogorov–Smirnov tests show a difference between the soluble and membrane helix angle distributions. The means of these distributions are similar, however, indicating that the differences measured in the test may be due to the very large number of short soluble helices compared with the small number of short membrane helices (see Figs. S6 and S7 in the Supporting Information).

These results indicate that helices of the same length should be compared when considering helix kinks. Throughout this work, membrane helices are compared with length-matched samples of the soluble helices.

Figure 1(b) shows the distribution of angles for the membrane set, and 50 length-matched samples from the soluble set. These two distributions are very similar. There are a similar number of helices with maximum angles <20° in the two datasets, and the two distributions peak at the same angle (10°). A few more membrane helices than soluble helices have angles between 20° and 30°, and a few more soluble helices than membrane helices have maximum angles above 50°. This is likely due to the membrane environment restricting the degree to which transmembrane helices can kink.

In order to investigate the properties of kinked helices, it is necessary to annotate helices as kinked or unkinked. As Figure 1 shows, the angle distribution is continuous, which is consistent with the lack of clear agreement in the literature on the ideal angle cut-off. Visual inspection of the angle distribution, and comparison to other methods,^17^–^19^ indicates that 20° is a reasonable choice. Using this angle, in the length matched sets, 32% of soluble helices are kinked, and 40% of membrane helices are kinked. Altering this angle in the range of 15°–25° has only a small effect on the results discussed below.

### Amino acid patterns around kinks

To investigate if the residue patterns around kinks in soluble and membrane helices are similar, we have examined the amino acid propensities in positions close to the kink, using length-matched sets of membrane and soluble helices. The propensities are calculated using sequence homologs.

**Figure 2:**
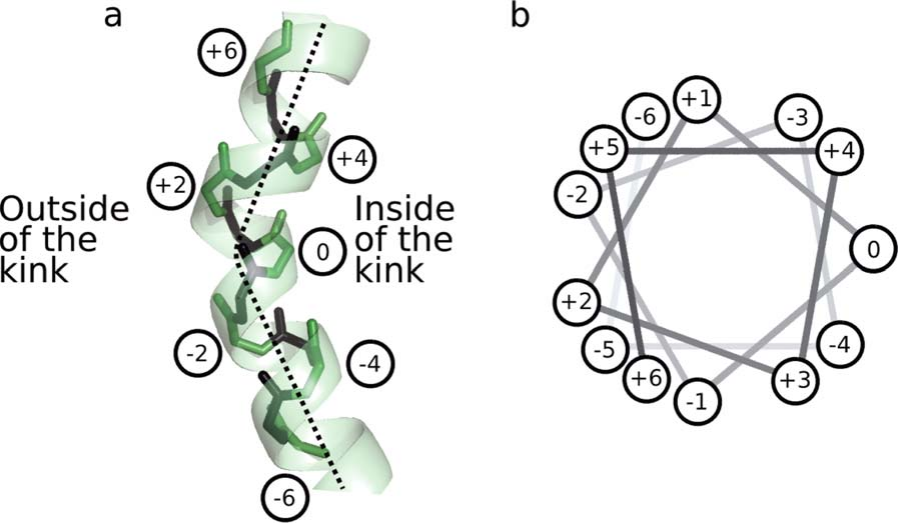
Kink numbering. (a) Numbering for an example kink, with even numbered residues (*green*) labeled. The *broken line* shows an approximate helix axis and is in the plane of the page. (b) A standard helical wheel diagram, showing the helix in (a). Kinks are numbered from N-terminal to C-terminal, with the kink residue given number 0. This scheme results in residues −4, −3, 0, +3, and +4 being on the inside of the kink, while residues −5, −2, +2, and +5 are on the outside. [Color figure can be viewed in the online issue, which is available at wileyonlinelibrary.com.]

Our kink identification method (described above) chooses a kink residue on the inside of the kink, as shown in Figure 2. The residues around each kink are numbered relative to this kink residue, with the kink residue as number 0, those residues toward the N-terminus as negative numbers, and those toward the C-terminus as positive numbers. Thus, position −5 is five residues from the kink residue, toward the N-terminus. A consequence of this numbering scheme is that some positions are consistently on the outside of the kink (e.g., −2 and +2), and some positions are consistently on the inside of the kink (e.g., −4, 0, and +4).

#### Proline

**Figure 3:**
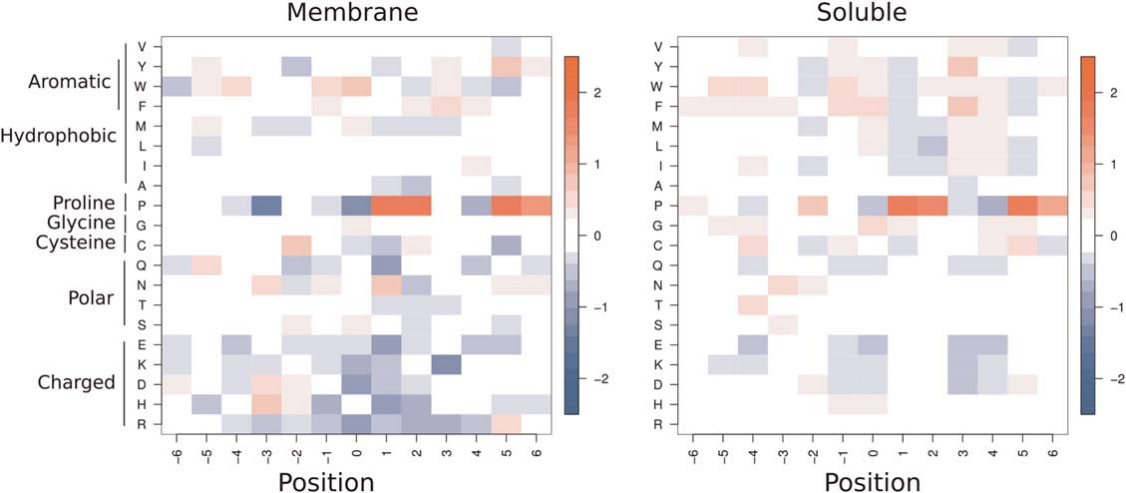
Amino acid propensities for membrane (left) and soluble (right) kinks. Each value is the propensity for a given residue to be at a given position relative to a kink. These are calculated using sequence profiles. Position 0 is the kink residue, position +1 is one residue toward the C-terminus from the kink residue, as shown in Figure 2. *Red* indicates positive propensities, while *blue* indicates negative propensities. Residues with positive propensities are found more frequently at that position in kinked helices than in helices in general. See Supporting Information Figure S4 for the analogous figure based on the MC-Helan analysis.

Figure 3 shows the amino acid propensities around kinks (≥20°) in the membrane and length-matched soluble helix sets. For both membrane and soluble helix kinks, Proline is present most strongly at position +2 (two residues C terminal to the kink, see Fig. 2), but also at other positions: +1, +5, and +6. These are all positions on the *outside* of the kink. Conversely it is disfavored at positions 0 and +4, the *inside* of the kink. In soluble kinks Proline is also slightly favored at positions before the kink (e.g., −2, −3, and −6), which are also the outside of the kink.

Our analysis has revealed that in both types of helices Proline typically occurs on the outside of kinks. This is due to both the physical size of its ring, and its lack of amide proton precluding the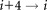 backbone hydrogen bond. Both of these factors tend to lengthen the distance between the *i*th residue (Proline) and the *i* – 4th residue, which causes the helix to kink away from the side of the helix containing Proline.

Proline is clearly very important to kinks. Approximately half of our membrane helices contain a Proline, compared with only one-third of the length-matched soluble helix sets (Table S3 in the Supporting Information). In terms of its ability to disrupt the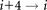 backbone hydrogen bond, Proline must be at least four residues from the N-terminus of the helix. Sixteen percent of transmembrane helices, and on average 15% of the length-matched soluble helix sets contain a Proline only in the first four residues. In these helices, we do not expect Proline to cause a kink. Classing these helices with Prolines only in the first four residues as non Proline containing, 95% of soluble helices, and 85% of membrane helices that contain Proline are kinked.

These Prolines are not necessarily close to the kink position identified above, but this trend does indicate that presence of Proline is likely to be linked to helix kinking. For comparison, however, not all kinked helices contain Proline: 32% of kinked transmembrane helices contain no Proline, and a clear majority (58%) of (length matched) kinked soluble helices do not contain a Proline. Additionally, there is a large proportion of kinked helices in both sets that do not contain a Proline even in the homologous sequence alignment.

#### Other amino acids

For other residues, the patterns in Figure 3 are less obvious, but those discussed here are seen in the results of both the Kink Finder and MC-Helan analyses. In soluble helices, Glycine is over-represented on the inside of kinks (positions 0, +1, +4, +5; see Fig. 2), and slightly over-represented at position 0 in membrane helix kinks. This may be due to the flexibility of Glycine, or the lack of a sidechain allowing the helix to bend toward the Glycine.

Serine is overrepresented in positions before the kink (−2 and 0 for membrane kinks, and −3 for soluble kinks), as is Asparagine (position −3 for both soluble and membrane). The aromatic residues (Phenylalanine, Tyrosine, and Tryptophan) show some clear periodic patterns in the soluble propensities, being over-represented at positions −5, −4, −1, 0, +3, and +4. There are similar but weaker patterns in the membrane propensities—for example, Phenylalanine is over-represented at positions +2, +3, and +4. Although it might be expected that large residues would be found on the outside of kinks due to their size, the aromatic residues are more frequently found on the inside of kinks. This suggests that there may be some pi-stacking interactions that stabilize kinks.

There is a hydrophobicity pattern in the soluble propensities, where we see small positive propensities from many hydrophobic residues at −4, 0, +3, and +4 (positions on the inside of the helix kink), and corresponding negative propensities for charged residues at these positions. This suggests that kinks in soluble protein helices favor having charged residues on the outside of the kinks, and hydrophobic residues on the inside of the kink (Fig. 2).

### Hydrogen bonds

Many of these residue patterns are thought to be due to the role of hydrogen bonds in kinks. While Proline cannot form hydrogen bonds from its amide, other residues, like Serine and Threonine, may stabilize kinked helices which lack some backbone hydrogen bonds. We find that broken hydrogen bonds are equally common in the membrane and length-matched soluble sets of helices (12.8% and 11.1% of residues in kinked membrane and soluble helices, respectively), and equally more common in the 13 residues around kinks (15.6% and 13.5% of residues in membrane and soluble kinks, see Table S4 in the Supporting Information). Broken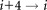 backbone hydrogen bonds are more frequent in kinks, and particularly on the outside of kinks (data not shown). Conversely,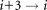 and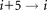 bonds are more frequent around kinks, suggesting that there is a general disruption of the normal α-helical structure around kinks. These are more frequent in positions around the kink where more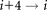 backbone hydrogen bonds are broken, indicating that they act to compensate for the loss of the normal backbone hydrogen bonds in kinked helices. However, there is no increase in frequency of sidechain to backbone hydrogen bonds around kinks.

### Motifs

Previous studies have suggested a number of sequence motifs which may cause kinks. Some of these have been suggested from observation in structures (e.g., Refs.^5^, ^19^, ^24^), while others have been identified from conserved sequence patterns (e.g., Refs.^31^–^32^). The occurrence of these motifs, and others we have identified, in both kinked and straight helices are shown in Table 1. This analysis uses only the sequences from the helix structures. Relevant motifs should be both frequently observed in kinks, and also more frequently observed in kinks than comparable segments of straight helices. Motifs that are present in more than 10% of kinks, and motifs that are present more than twice as frequently in kinked helices as compared with straight helices are highlighted in bold in Table 1. We have restricted the search for motifs to the 13 residues around a kink (positions −6 to +6 in Fig. 2). This allows a comparison to a background distribution of motifs in 13 residue sections of straight helices. The choice of a window means that longer motifs are less likely to be found than shorter ones (e.g., P is more frequently found that xxxxP). This is true for both the kinked and straight sections, so we can study both the frequency of a motif and its enrichment ratio (i.e., comparing the number of times a motif is observed in kinked and straight helices).

**Table 1:** Table of Amino Acid Motif Frequency in Kinks and Randomly Selected Parts of Straight Helices

Motif	Membrane			Soluble		
	% of kink segments that contain the motif	% of straight segments that contain the motif	Enrichment Ratio	% of kink segments that contain the motif	% of straight segments that contain the motif	Enrichment Ratio
[AVILMFYW]xxxP^5^	**38.5**	0.8	**47.3**	**12.7**	0.1	**200.4**
xxxxP	**58.5**	1.6	**37.2**	**36.7**	0.1	**250.9**
[ST]P^19^,^24^	5.3	0.0	**197.4**	3.4	0.2	**21.3**
[DR]P^13^	1.5	0.3	**4.4**	3.8	0.1	**39.8**
xP	**60.9**	6.4	**9.5**	**39.0**	1.7	**23.4**
xxxP	**58.5**	2.0	**28.7**	**37.3**	0.2	**170.5**
[AVILMFYW]xP^5^	**46.1**	2.8	**16.4**	**25.0**	0.4	**59.9**
GxP	3.9	0.2	**16.7**	1.4	0.0	**49.6**
xxP	**60.0**	3.5	**17.2**	**38.0**	0.7	**55.5**
P[ST]^8^,^24^	4.5	0.5	**8.7**	2.7	0.4	**6.1**
Px	**55.3**	9.6	**5.7**	**35.7**	4.6	**7.8**
P	**61.3**	9.9	**6.2**	**40.1**	4.6	**8.8**
[VALT]LWx[AG]YP^31^	0.2	0.0	NA	0.0	0.0	NA
GHPxVY[FI]^31^	0.0	0.0	NA	0.0	0.0	NA
[ST][ST]^5^,^30^	**10.7**	10.9	1.0	9.7	8.2	1.2
[ST]xx[ST]^30^	10.0	10.5	0.9	8.5	7.1	1.2
[ST]xxx[ST]^30^	8.5	9.5	0.9	7.7	7.0	1.1
GxxGxxxG^19^	1.3	1.0	1.3	0.0	0.1	0.2
GxxxGxxG^19^	0.8	0.9	0.9	0.2	0.0	**5.4**
[FYW]xxxSxxx[FYW]^19^	0.2	0.3	0.6	0.3	0.2	1.8
LSAxF^19^	0.0	0.3	0.0	0.1	0.0	NA
WLF[ST]^32^	0.4	0.0	NA	0.0	0.0	NA
[FYW]xxx[FYW]	**22.6**	11.7	1.9	9.6	6.4	1.5
[FYW]xxx[FYW]xx[FYW]	6.2	0.6	**10.8**	0.9	0.6	1.5
[FYW]xxx[FYW]xxx[FYW]	4.5	0.9	**4.8**	0.9	0.4	**2.2**
[FYW]xx[FYW]xxx[FYW]	3.6	1.2	**3.0**	1.2	0.4	**2.7**
[RHDEK]xxx[RHDEK]	7.9	8.9	0.9	**49.0**	57.7	0.8
[ASG]xxx[ASG]	**38.0**	47.8	0.8	**26.4**	32.4	0.8
[STNQ]xxx[STNQ]	**19.4**	17.5	1.1	**25.5**	25.2	1.0
G_0_ or G_+1_	**15.6**	17.8	0.9	**10.2**	6.6	1.5

Except those that involve Proline, very few of the motifs are observed in more than 10% of kinks. Similarly, few motifs are seen more than twice as frequently in kinks compared with straight helices (Table 1). Motifs that contain Proline are generally no more enriched in kinks than the corresponding sequence length containing just Proline. For example, the GxP motif has an enrichment ratio of 16.7 (i.e., it is found 16.7 times more frequently in kinks than straight helices), and the xxP motif has an enrichment ratio of 17.2. The [ST]P motif is an exception (found in 5% of membrane kinks), being very rarely in sections of straight membrane helices (enrichment ratio 197). Unlike the [ST]P motif, motifs containing Serine/Threonine without Proline (e.g., [ST][ST]) are not seen more frequently in kinks than in straight helices.

It has been suggested in some studies that small residues (particularly Glycine) allow flexibility in helices.^5^,^17^,^19^ The soluble propensities show Glycine as overrepresented at positions 0, +1, +4, and +5, suggesting that there may be motifs such as GxxxG present in kinks. However, we have found no small-residue motifs that occur more frequently in kinks than in straight helices.

As described in the previous section, aromatic residues had a high propensity to be around helix kinks. We find the [FYW]xxx[FYW] motif to be slightly enriched for both membrane and soluble kinks. It occurs in 23% of membrane kinks, compared with only 12% of straight membrane helices. Although this motif is less frequent in soluble kinks (in 10% of kinks), it is similarly more frequent than in the straight soluble helices (6%). Finally, we note that many of the remaining motifs are found in very few structures in our set, which prevents us from assessing if they are associated with kinks.

### Hydrophobicity and solvent accessible surface area

**Figure 4:**
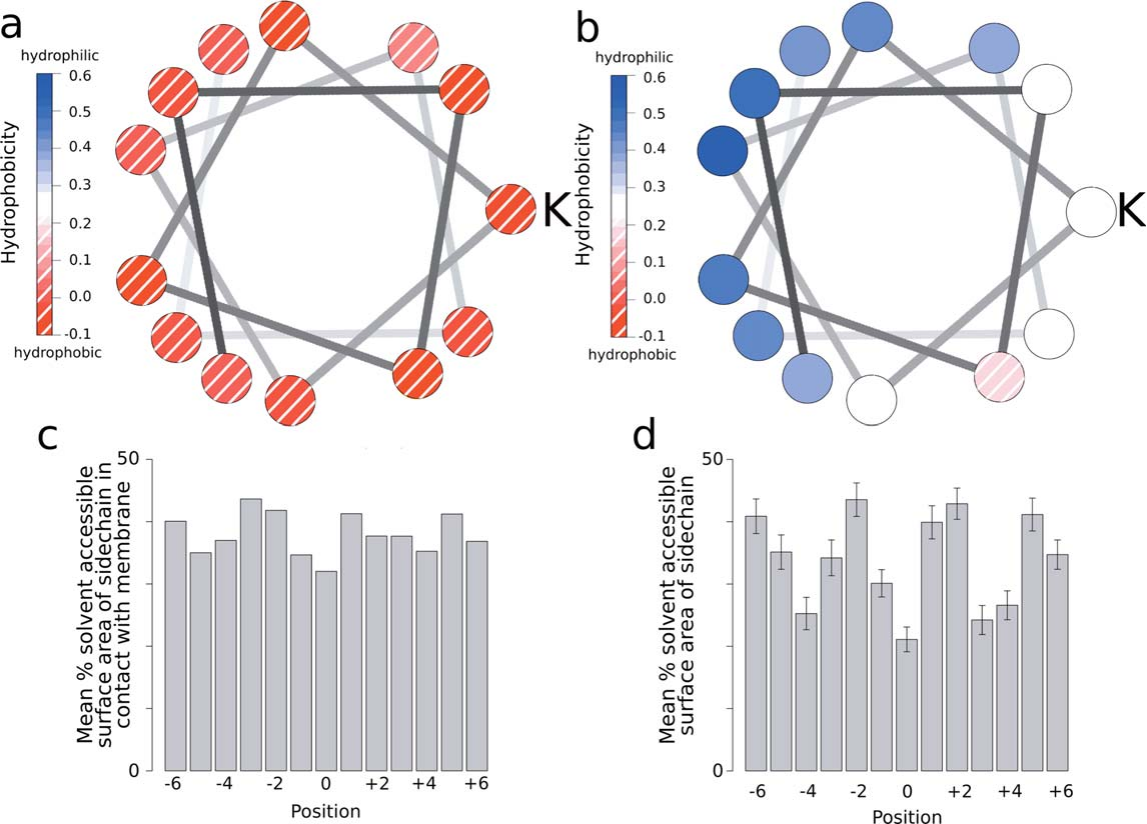
Hydrophobicities, solvent accessible surface areas, and membrane contacts. (a) Helical wheel diagram showing the average hydrophobicity of residues around membrane kinks. K indicates the kink residue (position 0 in Fig. 2). (b) Wheel diagram for soluble kinks. K indicates the kink residue. (c) Average percentage of residues in contact with the membrane in membrane kinks. (d) Average solvent accessible percentage of residues in soluble kinks. Bars show 2 s.d. from 50 samples. See Figure S5 in the Supporting Information for the analogous figure based on the MC-Helan analysis. [Color figure can be viewed in the online issue, which is available at wileyonlinelibrary.com.]

The amino acid propensities around kinks in Figure 3 show a periodic pattern. Hydrophobic residues are more frequently observed in positions on the inside of kinks (−4,0,+4), while charged and polar residues are less frequent at these positions. The mean hydrophobicity of each position in membrane and soluble kinks are shown in Figure 4. The hydrophobicities are calculated from the sequence profiles, derived from the homologous sequences. There is a clear pattern in the hydrophobicity of soluble kinks, with more hydrophobic residues on the inside of kinks, and more hydrophilic residues on the outside of kinks. This pattern is repeated in the fraction of residues which are solvent accessible, with residues on the outside of kinks being more solvent accessible than those the inside [Fig. 4(d)]. This indicates that soluble kinks point into the aqueous environment, meaning that the residues on the outside of the kink will be in the solvent (Fig. 2). The solvent accessible surface areas are calculated only from the structures.

This pattern of hydrophobicity and solvent accessible surface area may be related to the effect of solvent on hydrogen bonds in helices. Backbone hydrogen bonds on the hydrophilic side of amphipathic helices are generally longer than those on the hydrophobic side^54^ so that helices curve, or kink, away from the solvent side. This is due to the other available hydrogen bond donors and acceptors on the hydrophilic side of the helix (e.g., sidechains or solvent), which can form bonds to the backbone groups, withdrawing electron density from the backbone hydrogen bonds, causing them to lengthen. As expected, this hydrophobicity pattern is not repeated in the membrane kinks, as in this case the helix is surrounded by the rest of the protein or lipid.

## DISCUSSION

Our investigation has shown that kinked helices are not particular to membrane proteins. If soluble helices with the same length distribution as membrane helices are considered, a similar number of kinks are seen in membrane and soluble helices. Compared with membrane helix kinks, there are more soluble kinks with larger angles, which may be due to the membrane environment restricting the degree to which a helix can kink. This overall similarity of kinks in soluble and membrane helices is independent of the method used to assess their occurrence. Kink residues in this study are identified such that they are in a geometrically similar place with respect to the kink. However, since no other method takes the geometry of the kink into account when selecting the kink residue, the results reported here will not necessarily agree with those in earlier studies.

Proline is dominant in both membrane and soluble helix kinks, although there are more Prolines incorporated into long membrane helices than long soluble helices. Excluding the first four residues of the helix, the vast majority of Proline containing helices are kinked, but there are many kinked helices that do not contain Proline. The residue patterns around kinks are dominated by Proline, both for membrane and soluble kinks. The consistent choice of kink residue relative to the helix shape (the kink residue is on the inside of the kink), reveals that Proline occurs on the outside of the helix kinks. This consistent positioning reveals a number of other patterns—Glycine is favored on the inside of the kink, Serine is favored before and on the outside of the kink, and aromatic residues are favored on the inside of kinks. These patterns are observed in both the membrane and soluble helix sets.

Motifs containing aromatic residues are more frequently observed in kinks than straight helices, both in soluble and membrane proteins. Particularly, the [FYW]xxx[FYW] is a factor of 1.9 more frequent in membrane kinks than in straight membrane helices, and is found in 23% of membrane helix kinks. Many other motifs highlighted in previous research are either seen very infrequently or no more frequently than in straight helices. Allowing for the expected differences in residue frequencies in the two types of helix, the amino acid patterns around helix kinks are very similar in membrane and soluble proteins.

A strong hydrophobicity pattern is observed in soluble kinks—where solvent accessible and hydrophilic residues are seen on the outside of kinks, indicating that soluble kinks point into the solvent. While there is no hydrophobicity pattern in the membrane kinks, there is a tendency for aromatic residues to be observed on the inside of membrane kinks.

In this study, we have used relatively relaxed parameters to create the dataset, compared with some other studies.^16^,^19^ Making these parameters more strict reduces the available data, particularly in the membrane helix set. Changing the parameters, for example reducing the R-factor threshold, the maximum resolution threshold, or the redundancy cut-off, does not change the patterns observed (see Supporting Information Figs. S9–S11).

Overall, these results show that soluble helices equally likely to be kinked as their membrane counterparts, and that these soluble kinks point into the solvent. This suggests that kinks are an important structural feature of soluble proteins and may, like their membrane counterparts, be functionally important.
