# Supplementary material for: Helix kinks are equally prevalent in soluble and membrane proteins
Source: Proteins. 2014 Apr 16;82(9):1960–70. doi: 10.1002/prot.24550 (PMC4285789; doi:10.1002/prot.24550)
Supplement: Supplementary file 1 [file prot0082-1960-SD1.pdf]

Helix kinks are equally prevalent in soluble and membrane proteins. Supplementary Information

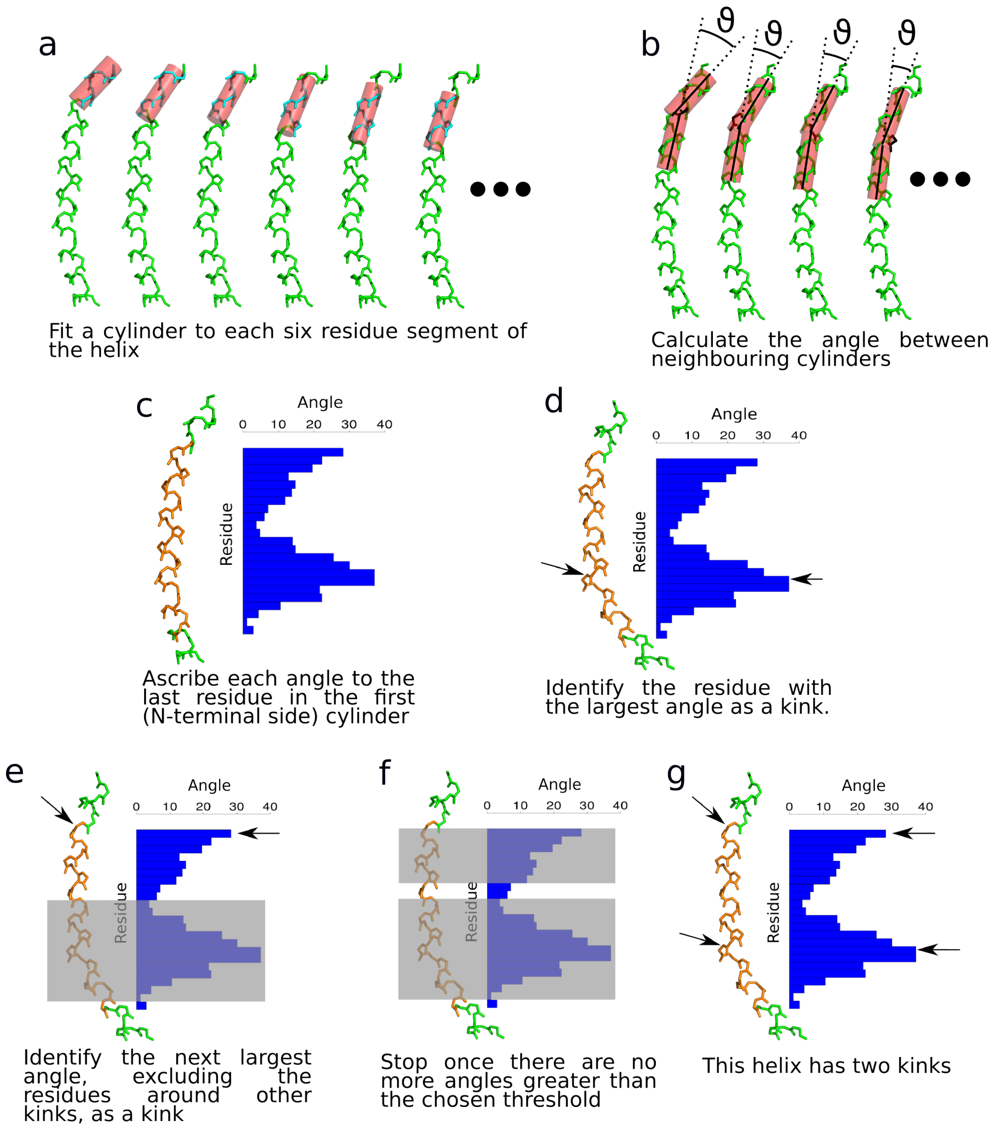

Figure S1. Kink Finder algorithm. (a) Axes are fitted to the backbone atoms of a sliding window of six residues. (b) and (c) These axes are used to calculate a local angle for each residue. (d) The residue with the largest angle is annotated as kinked (e) Additional kinks are identified at residues where the angle is $\geq 20°$, the residue is at least four residues from another kink, and there is at least one residue with a local angle $\leq 10°$ the residue and all other kinks in the helix. The method for choosing the kink residue for each kink is shown in Figure S2.

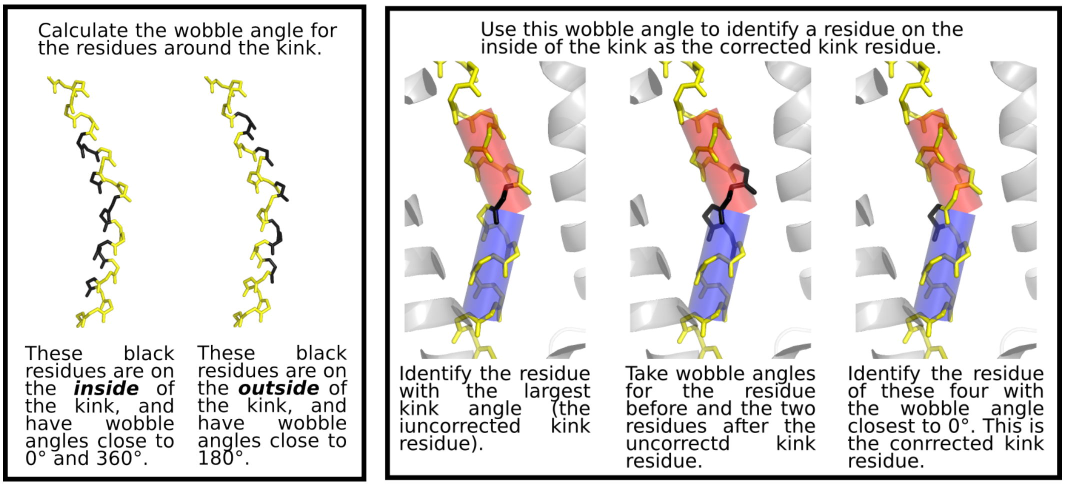

Figure S2. Kink Finder's choice of kink residue. Figure S3 explains the specific method used to determine the wobble angle.

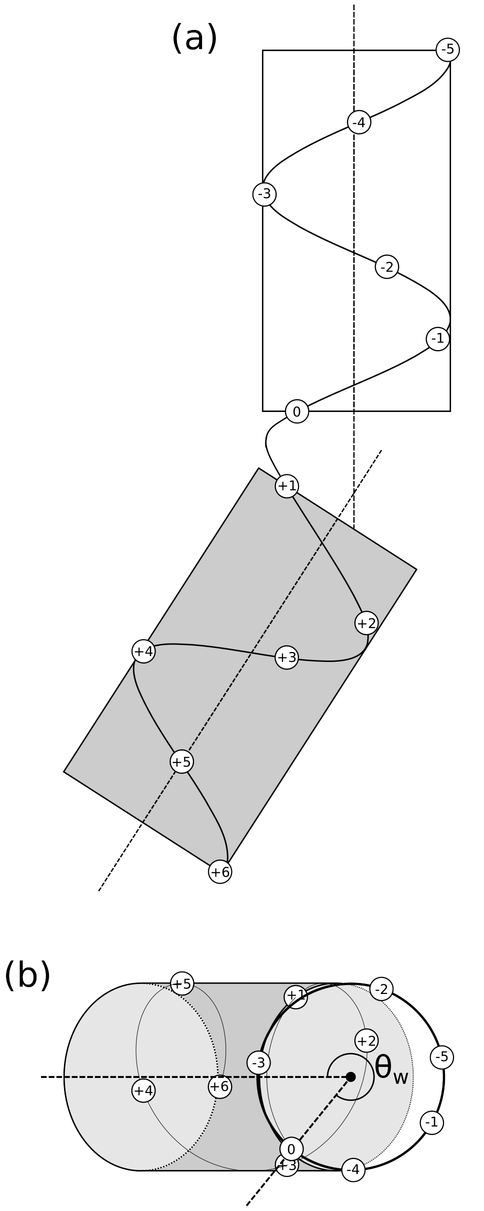

Figure S3. Calculating the wobble angle (a) Representation of a kinked helix with fitted cylinders. Only Cα atoms are shown, lettered sequentially -5 (N-terminus) to +6 (C-terminus). The fitted axes are shown by the dotted lines, and are in the plane of the page. The N-terminal cylinder is white, and the C-terminal cylinder is grey. (b) the same representation, rotated to look down the N-terminal axis. Atom 0 is in the plane of the page, and the C-terminal axis has been projected onto this plane. The wobble angle for each Cα atom is the angle between this vector, and the vector perpendicular to the n-terminal axis which passes through the Cα atom. θ_w is the wobble angle for atom 0. The wobble angle is used to select the kink residue, as shown in Figure S2.

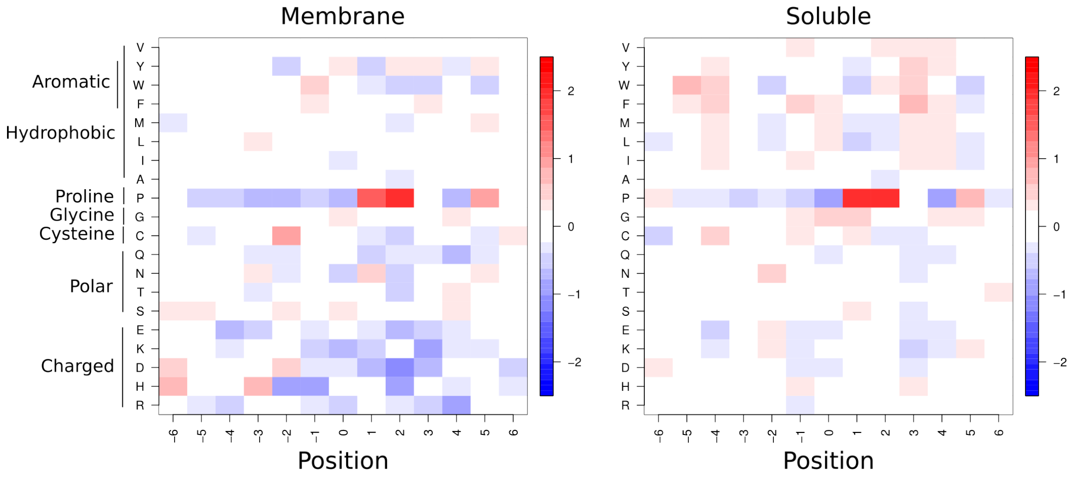

Figure S4. Amino acid propensities for membrane (left) and soluble (right) kinks, identified by MC-Helan. Each value is the propensity for a given residue to be at a given position relative to a kink. These are calculated using sequence profiles. Position 0 is the kink residue, position +1 is one residue towards the C-terminus from the kink residue, as shown in Figure 2. Red indicates positive propensities, while blue indicates negative propensities. Residues with positive propensities are found more frequently at that position in kinked helices than in helices in general.

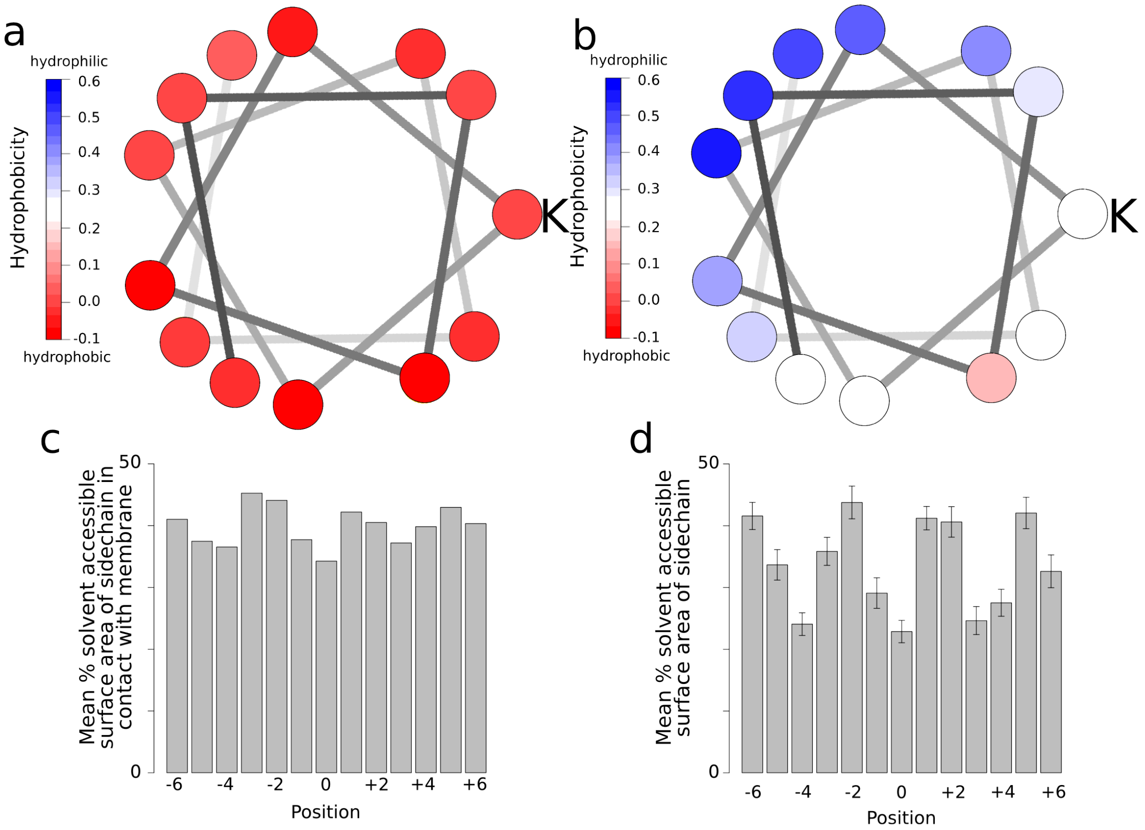

Figure S5. Hydrophobicities, solvent accessible surface areas, and membrane contacts for kinks identified by MC-Helan. (a) Standard helical wheel diagram showing the average hydrophobicity of residues around membrane kinks. K indicates the kink residue (position 0 in Figure 2). (b) Standard helical wheel diagram for soluble kinks. K indicates the kink residue. (c) Average percentage of residue in contact with the membrane in kinks. (d) Average solvent accessible percentage of residues in soluble kinks. Bars show 2 s.d. from 50 length-matched samples.

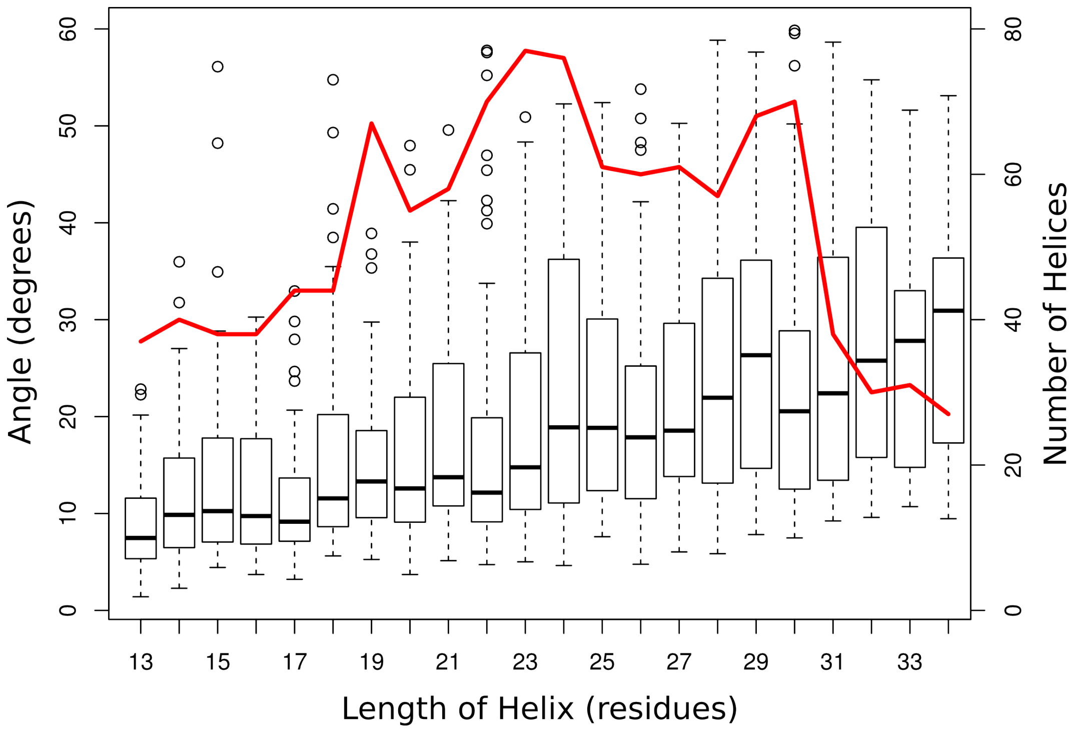

Figure S6. Lengths and angles of membrane helices. Plot of maximum kink angles (calculated by Kink Finder) for each different length of helices. Each boxplot shows the distribution of maximum angle in each helix of that length (scale on left axis). The red line shows the total number of helices of each length (scale on right axis). Note how the maximum angle increases as the helices get longer, and that the majority of the helices are between 20 and 30 residues long.

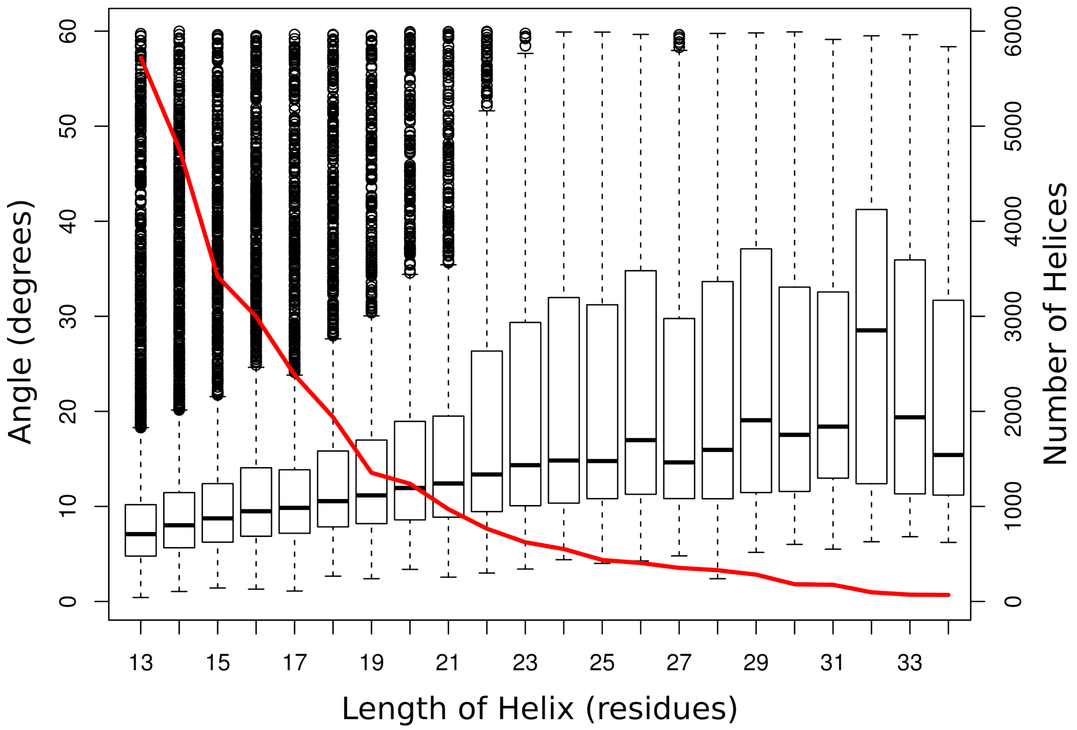

Figure S7. Lengths and angles of soluble helices. Plot of maximum kink angles (calculated by Kink Finder) for each different length of helices. Each boxplot shows the distribution of maximum angle in each helix of that length (scale on left axis). The red line shows the total number of helices of each length (scale on right axis). Note how the maximum angle increases as the helices get longer, and that the majority of the helices are fewer than 20 residues long.

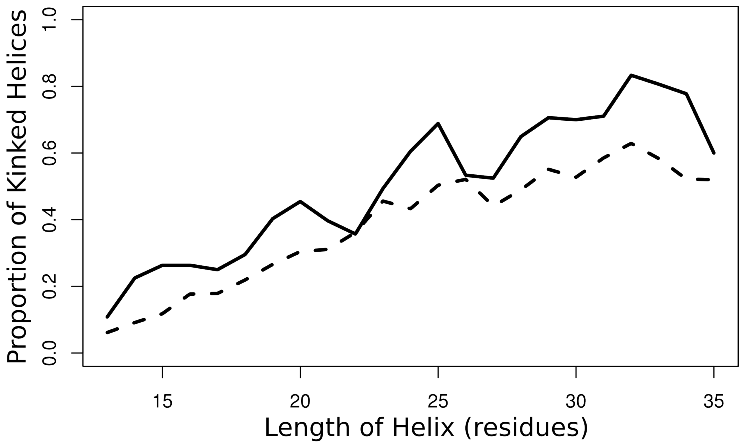

Figure S8. Proportion of kinked helices, as determined by MC-Helan. The proportion of helices of a given length that are kinked (with an angle over 12°) is similar for membrane (solid line) and soluble (dashed line) proteins. In the same way that the maximum kink angle in each helix (as defined by Kink Finder) increases as the helix gets longer, the probability of a helix being kinked (as defined by MC-Helan) increases as the helix gets longer.

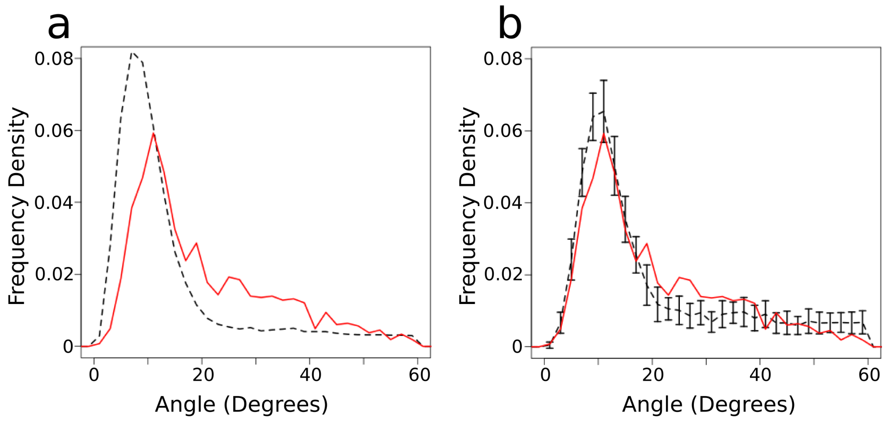

Figure S9. Reproduction of Figure 1, for data sets with thresholds: 80% sequence identity, resolution < 5Å, R-factor < 0.9. (a) The distribution of maximum angles in all membrane (solid red) and all soluble (dashed black) helices. (b) The distribution of maximum angles in length matched sets of membrane and soluble helices. The soluble values are means of 50 samples which are matched to the same length distribution as the membrane helices. Again, the solid red line shows the membrane distribution, while the dashed black line shows the soluble distribution. Bars show 2 s.d. from 50 samples.

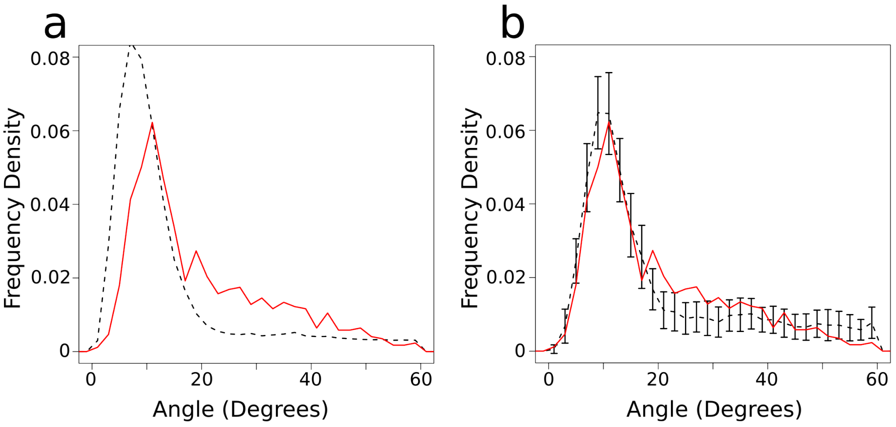

Figure S10. Reproduction of Figure 1, for data sets with thresholds: 80% sequence identity, resolution < 3Å, R-factor < 0.9. (a) The distribution of maximum angles in all membrane (solid red) and all soluble (dashed black) helices. (b) The distribution of maximum angles in length matched sets of membrane and soluble helices. The soluble values are means of 50 samples which are matched to the same length distribution as the membrane helices. Again, the solid red line shows the membrane distribution, while the dashed black line shows the soluble distribution. Bars show 2 s.d. from 50 samples.

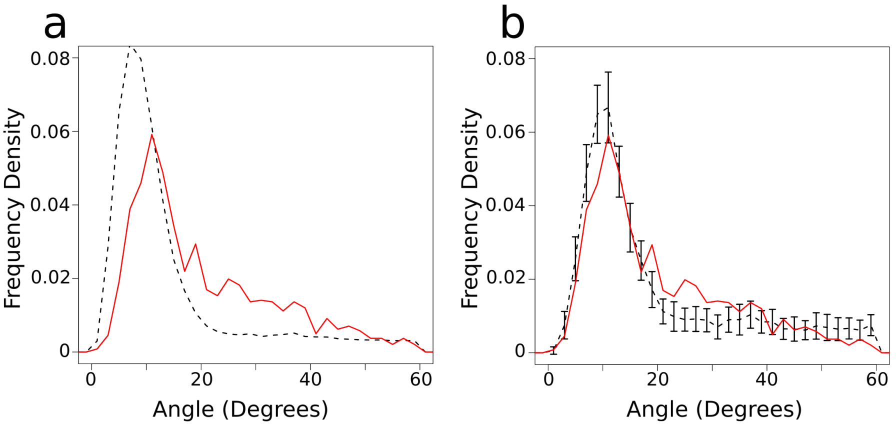

Figure S11. Reproduction of Figure 1, for data sets with thresholds: 50% sequence identity, resolution < 5Å, R-factor < 0.9. (a) The distribution of maximum angles in all membrane (solid red) and all soluble (dashed black) helices. (b) The distribution of maximum angles in length matched sets of membrane and soluble helices. The soluble values are means of 50 samples which are matched to the same length distribution as the membrane helices. Again, the solid red line shows the membrane distribution, while the dashed black line shows the soluble distribution. Bars show 2 s.d. from 50 samples.

Table S1. Amino Acid Motif Frequencies

	Membrane			Soluble		
Motif	% Kinks	% Straight Helices	Ratio	% Kinks	% Straight Helices	Ratio
[AVILMFYW]xxxP	**27.2**	0.6	**49.3**	9.5	0.0	**453.4**
xxxxP	**41.9**	1.0	**43.6**	**27.7**	0.1	**344.3**
[ST]P	3.7	0.0	**81.5**	2.4	0.2	**12.8**
[DR]P	0.8	0.2	**3.4**	2.6	0.2	**17.4**
xP	**44.3**	7.0	**6.4**	**29.4**	2.0	**14.9**
xxxP	**42.5**	1.8	**23.9**	**28.1**	0.2	**172.1**
[AVILMFYW]xP	**32.1**	3.1	**10.4**	**19.1**	0.4	**46.5**
GxP	2.9	0.0	**128.0**	0.9	0.0	**36.3**
xxP	**43.8**	3.5	**12.4**	**28.7**	0.7	**38.8**
P[ST]	4.1	0.1	**60.1**	2.5	0.6	**4.5**
Px	**43.3**	11.0	**3.9**	**30.0**	5.5	**5.5**
P	**45.0**	11.3	**4.0**	**30.6**	5.5	**5.6**
[VALT]LWx[AG]YP	0.3	0.0	NA	0.0	0.0	NA
GHPxVY[FI]	0.3	0.0	NA	0.0	0.0	NA
[ST][ST]	**12.6**	9.8	1.3	9.4	7.3	1.3
[ST]xx[ST]	9.9	10.8	0.9	9.1	7.0	1.3
[ST]xxx[ST]	9.3	7.8	1.2	8.7	6.8	1.3
GxxGxxxG	1.1	1.3	0.8	0.1	0.0	**2.1**
GxxxGxxG	1.1	0.6	1.8	0.1	0.0	**5.2**
[FYW]xxxSxxx[FYW]	0.7	0.4	1.9	0.3	0.1	**3.2**
LSAxF	0.3	0.3	0.9	0.1	0.0	**6.0**
WLF[ST]	0.3	0.0	NA	0.0	0.0	NA
[FYW]xxx[FYW]	**17.1**	12.7	1.3	9.0	5.9	1.5
[FYW]xxx[FYW]xx[FYW]	2.9	0.2	**13.7**	1.1	0.4	**2.4**
[FYW]xxx[FYW]xxx[FYW]	3.6	0.4	**8.0**	1.0	0.4	**2.7**
[FYW]xx[FYW]xxx[FYW]	2.1	1.1	2.0	1.0	0.4	**2.6**
[RHDEK]xxx[RHDEK]	8.5	9.5	0.9	**50.6**	58.5	0.9
[ASG]xxx[ASG]	**39.7**	47.0	0.8	**26.3**	33.3	0.8
[STNQ]xxx[STNQ]	**17.6**	18.0	1.0	**25.1**	24.7	1.0
G_0 or G_{+1}	**16.0**	19.8	0.8	**10.9**	6.2	1.7

Table of amino acid motif frequency in kinks and randomly selected parts of straight helices, using the kinks identified by MC-Helan. Motifs that occur in more than 10% of kinks, or are more than twice as frequent in kinks than straight helices are highlighted in bold. First section: Proline containing motifs. Second section: motifs highlighted by other authors. Third section: aromatic and polar motifs. Fourth section: small residue motifs.

Table S2. P Values for K-S Tests

Helix Length (residues)	p value
13	3.9×10^{-07}
14	0.0004
15	0.0002
16	0.003
17	0.0006
18	0.317
19	0.303
20	0.174
21	0.866
22	0.018
23	0.212
24	0.757
25	0.917
26	0.329
27	0.323
28	0.084
29	0.033
30	0.246
31	0.315
32	0.113
33	0.594
34	0.335
35	0.758
36	0.667
37	0.429
38	0.016
39	0.900
40	0.800

P values for Kolmogorov-Smirnov tests on the distribution of maximum kink angles in helices of given lengths. P values ≤ 0.05 indicate that the distributions of angles for soluble and membrane helices (of a given length) are different.

Table S3. Prolines in Helices

	Membrane		Soluble length-matched	
	Kinked	Straight	Kinked	Straight
Proline in helix	29.6	18.3	17.0	12.2
No Proline in helix	9.9	42.3	15.0	55.7
Proline in profile	33.9	27.8	23.2	26.3
No Proline in profile	5.5	32.8	8.9	41.6
Proline in helix (5th and subsequent residues)	26.7	4.8	13.5	0.7
No Proline in helix (5th and subsequent residues)	12.7	55.8	18.5	67.3
Proline in profile (5th and subsequent residues)	31.1	10.3	18.9	5.4
No Proline in profile (5th and subsequent residues)	8.3	50.2	13.2	62.5

Percentage of kinked and non kinked Helices containing Proline, as identified by Kink Finder. Each 2x2 table shows the percentage of helices in each of 4 groups - kinked with Proline in the sequence (profile), kinked without Proline in the sequence (profile), straight with Proline in the sequence (profile), straight without Proline in the sequence (profile). The bottom half of the table ignores the first 4 (N-terminal) residues of helices when determining if there is a Proline in the sequence (profile), as these do not have the same effect on the helix structure as those later in the helix.

Table S4. Hydrogen Bonds

	Membrane			Soluble		
	Kinked Helices	Straight Helices	Kinks	Straight Helices	Kinked Helices	Kinks
Residues missing backbone hydrogen bond	15.6%	12.8%	6.7%	13.5%	11.1%	5.7%
Residues missing backbone hydrogen bond, excluding Prolines	14.6%	12.3%	6.3%	11.8%	9.7%	5.7%

Frequency of broken backbone hydrogen bonds in kinks and helices. Each value is the percentage of residues that have no mainchain to mainchain hydrogen bond to their carbonyl group. The second row excludes kinks and helices that contain Proline residues.

Table S5. Membrane Helices

Code	C	St.	End	Res.	R	Code	C	St.	End	Res.	R	Code	C	St.	End	Res.	R
1A91	A	2	38	NA	NA	1A91	A	47	78	NA	NA	1AIG	L	33	56	2.600	0.21
1AIG	L	84	111	2.600	0.21	1AIG	L	116	139	2.600	0.21	1AIG	L	171	198	2.600	0.21
1AIG	L	227	249	2.600	0.21	1AR1	B	26	58	2.700	0.21	1AR1	B	76	104	2.700	0.21
1BCC	D	199	227	3.160	0.27	1BCC	E	30	58	3.160	0.27	1BE3	C	34	51	3.000	0.26
1BE3	C	137	149	3.000	0.26	1BE3	C	172	200	3.000	0.26	1BE3	C	225	242	3.000	0.26
1BE3	C	287	307	3.000	0.26	1BE3	C	319	339	3.000	0.26	1BE3	C	345	376	3.000	0.26
1BE3	C	76	102	3.000	0.26	1BE3	C	110	130	3.000	0.26	1BE3	G	36	70	3.000	0.26
1BE3	J	18	46	3.000	0.26	1C17	M	101	125	NA	NA	1C17	M	137	166	NA	NA
1C17	M	202	228	NA	NA	1C17	M	235	263	NA	NA	1C8S	A	10	31	2.000	0.17
1C8S	A	38	61	2.000	0.17	1C8S	A	82	100	2.000	0.17	1C8S	A	202	221	2.000	0.17
1C8S	A	177	191	2.000	0.17	1C8S	A	106	127	2.000	0.17	1C8S	A	133	151	2.000	0.17
1DXR	H	12	35	2.000	0.19	1DXR	L	33	54	2.000	0.19	1DXR	L	84	111	2.000	0.19
1DXR	L	116	139	2.000	0.19	1DXR	L	171	198	2.000	0.19	1DXR	L	228	250	2.000	0.19
1DXR	M	53	76	2.000	0.19	1DXR	M	111	137	2.000	0.19	1DXR	M	143	166	2.000	0.19
1DXR	M	262	284	2.000	0.19	1DXR	M	198	225	2.000	0.19	1DXR	M	241	253	2.000	0.19
1E7P	C	76	97	3.100	0.28	1E7P	C	202	236	3.100	0.28	1E7P	C	22	46	3.100	0.28
1E7P	C	121	149	3.100	0.28	1E7P	C	171	193	3.100	0.28	1EHK	B	6	38	2.400	0.22
1EK9	A	23	36	2.100	0.21	1EK9	A	78	93	2.100	0.21	1EK9	A	229	242	2.100	0.21
1EYS	H	11	33	2.200	0.23	1EYS	L	33	55	2.200	0.23	1EYS	L	92	117	2.200	0.23
1EYS	L	124	147	2.200	0.23	1EYS	L	234	254	2.200	0.23	1EYS	L	179	205	2.200	0.23
1EYS	M	55	76	2.200	0.23	1EYS	M	112	137	2.200	0.23	1EYS	M	144	167	2.200	0.23
1EYS	M	263	283	2.200	0.23	1EYS	M	199	224	2.200	0.23	1EZV	C	32	51	2.300	0.22
1EZV	C	75	102	2.300	0.22	1EZV	C	173	202	2.300	0.22	1EZV	C	226	244	2.300	0.22
1EZV	C	288	308	2.300	0.22	1EZV	C	320	340	2.300	0.22	1EZV	C	348	379	2.300	0.22
1EZV	C	111	134	2.300	0.22	1EZV	C	138	150	2.300	0.22	1EZV	E	51	79	2.300	0.22
1EZV	G	61	81	2.300	0.22	1F88	B	34	63	2.800	0.19	1F88	B	71	98	2.800	0.19
1F88	B	108	137	2.800	0.19	1F88	B	153	166	2.800	0.19	1F88	B	201	223	2.800	0.19
1F88	B	247	276	2.800	0.19	1F88	B	291	308	2.800	0.19	1FX8	A	7	33	2.200	0.20
1FX8	A	41	59	2.200	0.20	1FX8	A	145	167	2.200	0.20	1FX8	A	178	193	2.200	0.20
1FX8	A	204	216	2.200	0.20	1FX8	A	234	253	2.200	0.20	1FX8	A	85	105	2.200	0.20
1H2S	B	24	49	1.930	0.23	1H2S	B	53	81	1.930	0.23	1IJD	A	15	35	3.000	0.24
1IJD	B	8	33	3.000	0.24	1J4N	A	6	34	2.200	0.27	1J4N	A	51	69	2.200	0.27
1J4N	A	94	115	2.200	0.27	1J4N	A	141	158	2.200	0.27	1J4N	A	170	184	2.200	0.27
1J4N	A	217	229	2.200	0.27	1JB0	J	13	32	2.500	0.20	1JB0	L	43	64	2.500	0.20
1JB0	L	74	99	2.500	0.20	1JB0	L	114	138	2.500	0.20	1KF6	C	106	128	2.700	0.23
1KF6	C	20	49	2.700	0.23	1KF6	C	67	89	2.700	0.23	1KF6	D	12	41	2.700	0.23
1KF6	D	99	115	2.700	0.23	1KF6	D	61	88	2.700	0.23	1KPL	B	33	65	3.000	0.26
1KPL	B	78	99	3.000	0.26	1KPL	B	128	141	3.000	0.26	1KPL	B	152	165	3.000	0.26
1KPL	B	215	232	3.000	0.26	1KPL	B	330	348	3.000	0.26	1KPL	B	444	456	3.000	0.26
1KPL	B	171	187	3.000	0.26	1KPL	B	253	283	3.000	0.26	1KPL	B	288	305	3.000	0.26
1KPL	B	357	377	3.000	0.26	1KPL	B	421	438	3.000	0.26	1KQF	B	248	277	1.600	0.18
1KQF	C	12	36	1.600	0.18	1KQF	C	50	75	1.600	0.18	1KQF	C	111	133	1.600	0.18
1KQF	C	145	174	1.600	0.18	1KZU	B	5	35	2.500	0.23	1L7V	A	3	32	3.200	0.26
1L7V	A	56	81	3.200	0.26	1L7V	A	93	106	3.200	0.26	1L7V	A	228	250	3.200	0.26
1L7V	A	276	295	3.200	0.26	1L7V	A	305	323	3.200	0.26	1L7V	A	114	138	3.200	0.26
1L7V	A	142	167	3.200	0.26	1L7V	A	190	213	3.200	0.26	1LGH	A	19	38	2.400	0.21
1LGH	B	10	41	2.400	0.21	1M56	A	26	55	2.300	0.24	1M56	A	92	115	2.300	0.24
1M56	A	136	154	2.300	0.24	1M56	A	186	213	2.300	0.24	1M56	A	227	257	2.300	0.24
1M56	A	273	305	2.300	0.24	1M56	A	313	326	2.300	0.24	1M56	A	342	370	2.300	0.24
1M56	A	450	476	2.300	0.24	1M56	A	491	520	2.300	0.24	1M56	A	381	400	2.300	0.24
1M56	A	414	444	2.300	0.24	1M56	B	49	82	2.300	0.24	1M56	B	98	127	2.300	0.24
1M56	C	17	36	2.300	0.24	1M56	C	42	67	2.300	0.24	1M56	C	73	106	2.300	0.24
1M56	C	197	228	2.300	0.24	1M56	C	238	259	2.300	0.24	1M56	C	134	155	2.300	0.24
1M56	C	161	187	2.300	0.24	1M56	D	19	49	2.300	0.24	1MM4	A	7	19	NA	NA
1N7L	A	25	50	NA	NA	1NEK	C	103	128	2.600	0.25	1NEK	C	22	51	2.600	0.25
1NEK	C	69	96	2.600	0.25	1NKZ	A	15	35	2.000	0.17	1OCC	A	12	41	2.800	0.20
1OCC	A	52	87	2.800	0.20	1OCC	A	95	115	2.800	0.20	1OCC	A	142	169	2.800	0.20
1OCC	A	184	212	2.800	0.20	1OCC	A	229	261	2.800	0.20	1OCC	A	270	283	2.800	0.20
1OCC	A	299	327	2.800	0.20	1OCC	A	407	433	2.800	0.20	1OCC	A	448	477	2.800	0.20
1OCC	A	336	359	2.800	0.20	1OCC	A	372	401	2.800	0.20	1OCC	B	15	45	2.800	0.20
1OCC	B	61	87	2.800	0.20	1OCC	C	73	105	2.800	0.20	1OCC	C	193	223	2.800	0.20
1OCC	C	234	254	2.800	0.20	1OCC	C	16	34	2.800	0.20	1OCC	C	40	65	2.800	0.20
1OCC	C	129	152	2.800	0.20	1OCC	C	156	183	2.800	0.20	1OCC	D	77	102	2.800	0.20
1OCC	G	13	37	2.800	0.20	1OCC	I	12	52	2.800	0.20	1OCC	K	13	34	2.800	0.20
1OCC	L	18	44	2.800	0.20	1OCC	M	12	41	2.800	0.20	1OCR	J	26	54	2.350	0.20
1ORQ	C	29	50	3.200	0.25	1ORQ	C	60	78	3.200	0.25	1ORQ	C	147	170	3.200	0.25
1ORQ	C	207	236	3.200	0.25	1PV6	A	7	35	3.500	0.29	1PV6	A	43	70	3.500	0.29
1PV6	A	168	185	3.500	0.29	1PV6	A	221	247	3.500	0.29	1PV6	A	254	284	3.500	0.29
1PV6	A	290	306	3.500	0.29	1PV6	A	312	338	3.500	0.29	1PV6	A	142	157	3.500	0.29
1PV6	A	345	374	3.500	0.29	1PV6	A	378	398	3.500	0.29	1PW4	A	20	52	3.300	0.30

Helices in membrane set. Code = PDB Code; C = Chain identifier; St. = Number of first residue in helix; End = Number of last residue in helix; Res. = Resolution (Angstroms); R = R-Factor. NAs in the last two columns indicate the protein structure was solved by a method other that X-ray diffraction.

Table S5 continued. Membrane Helices

Code	C	St.	End	Res.	R	Code	C	St.	End	Res.	R	Code	C	St.	End	Res.	R
1PW4	A	122	146	3.300	0.30	1PW4	A	154	178	3.300	0.30	1PW4	A	191	206	3.300	0.30
1PW4	A	253	276	3.300	0.30	1PW4	A	290	315	3.300	0.30	1PW4	A	321	338	3.300	0.30
1PW4	A	351	372	3.300	0.30	1PW4	A	382	408	3.300	0.30	1PW4	A	415	443	3.300	0.30
1PW4	A	65	78	3.300	0.30	1PW4	A	95	109	3.300	0.30	1Q90	A	249	280	3.100	0.22
1QLE	C	17	34	3.000	0.23	1QLE	C	54	74	3.000	0.23	1QLE	C	80	113	3.000	0.23
1QLE	C	206	232	3.000	0.23	1QLE	C	247	266	3.000	0.23	1QLE	C	141	163	3.000	0.23
1QLE	C	169	194	3.000	0.23	1QLE	D	17	46	3.000	0.23	1RC2	A	2	24	2.500	0.23
1RC2	A	36	53	2.500	0.23	1RC2	A	81	103	2.500	0.23	1RC2	A	131	151	2.500	0.23
1RC2	A	162	176	2.500	0.23	1RC2	A	200	224	2.500	0.23	1RH5	C	30	47	3.200	0.24
1RHZ	A	23	40	3.500	0.25	1RHZ	A	76	88	3.500	0.25	1RHZ	A	104	127	3.500	0.25
1RHZ	A	137	163	3.500	0.25	1RHZ	A	170	183	3.500	0.25	1RHZ	A	210	228	3.500	0.25
1RHZ	A	256	275	3.500	0.25	1RHZ	A	313	332	3.500	0.25	1RHZ	A	367	394	3.500	0.25
1RHZ	A	401	422	3.500	0.25	1RHZ	B	3	21	3.500	0.25	1RHZ	B	31	64	3.500	0.25
1S5L	x	2015	2043	3.500	0.30	1SQQ	K	8	35	3.000	0.23	1UYN	X	788	810	2.600	0.23
1VF5	D	23	42	3.000	0.26	1VRY	A	2	14	NA	NA	1VRY	A	34	51	NA	NA
1WP1	A	66	82	2.560	0.26	1WP1	A	131	147	2.560	0.26	1WP1	A	278	294	2.560	0.26
1WP1	A	339	355	2.560	0.26	1WRG	A	12	41	NA	NA	1XIO	A	3	26	2.000	0.23
1XIO	A	34	56	2.000	0.23	1XIO	A	71	91	2.000	0.23	1XIO	A	159	185	2.000	0.23
1XIO	A	195	223	2.000	0.23	1XIO	A	99	121	2.000	0.23	1XIO	A	125	153	2.000	0.23
1XL6	A	47	69	2.850	0.27	1XL6	A	108	135	2.850	0.27	1XME	A	11	45	2.300	0.22
1XME	A	65	97	2.300	0.22	1XME	A	105	125	2.300	0.22	1XME	A	143	172	2.300	0.22
1XME	A	181	212	2.300	0.22	1XME	A	221	255	2.300	0.22	1XME	A	262	274	2.300	0.22
1XME	A	292	326	2.300	0.22	1XME	A	347	368	2.300	0.22	1XME	A	380	408	2.300	0.22
1XME	A	415	444	2.300	0.22	1XME	A	463	492	2.300	0.22	1XME	A	527	551	2.300	0.22
1XRD	A	10	41	NA	NA	1YCE	a	3	44	2.400	0.20	1YCE	a	50	79	2.400	0.20
1YEW	A	185	207	2.800	0.27	1YEW	A	232	256	2.800	0.27	1YEW	B	14	43	2.800	0.27
1YEW	B	64	79	2.800	0.27	1YEW	B	90	104	2.800	0.27	1YEW	B	141	164	2.800	0.27
1YGM	A	33	54	NA	NA	1YQ3	D	5	29	2.200	0.17	1YQ3	D	33	58	2.200	0.17
1YQ3	D	62	86	2.200	0.17	1YST	H	12	34	3.000	0.23	1Z98	A	34	63	2.100	0.18
1Z98	A	74	92	2.100	0.18	1Z98	A	161	181	2.100	0.18	1Z98	A	199	213	2.100	0.18
1Z98	A	116	139	2.100	0.18	1Z98	A	236	262	2.100	0.18	1ZCD	A	13	29	3.450	0.30
1ZCD	A	59	83	3.450	0.30	1ZCD	A	156	174	3.450	0.30	1ZCD	A	181	199	3.450	0.30
1ZCD	A	223	236	3.450	0.30	1ZCD	A	247	271	3.450	0.30	1ZCD	A	358	370	3.450	0.30
1ZOY	D	98	121	2.400	0.23	1ZOY	D	38	62	2.400	0.23	1ZOY	D	66	91	2.400	0.23
1ZRT	D	221	249	3.500	0.30	1ZZA	A	13	34	NA	NA	1ZZA	A	64	81	NA	NA
2AXT	a	5036	5053	3.000	0.23	2AXT	a	5143	5165	3.000	0.23	2AXT	a	5269	5294	3.000	0.23
2AXT	a	5111	5136	3.000	0.23	2AXT	a	5196	5221	3.000	0.23	2AXT	z	5003	5028	3.000	0.23
2AXT	z	5037	5061	3.000	0.23	2B2F	A	3	27	1.720	0.18	2B2F	A	85	104	1.720	0.18
2B2F	A	187	208	1.720	0.18	2B2F	A	214	241	1.720	0.18	2B2F	A	246	260	1.720	0.18
2B2F	A	269	292	1.720	0.18	2B2F	A	336	366	1.720	0.18	2B2F	A	35	53	1.720	0.18
2B2F	A	114	136	1.720	0.18	2B6P	A	10	31	2.400	0.24	2B6P	A	39	59	2.400	0.24
2B6P	A	83	107	2.400	0.24	2B6P	A	127	148	2.400	0.24	2B6P	A	160	174	2.400	0.24
2BG9	A	406	432	4.000	NA	2BG9	A	219	237	4.000	NA	2BG9	A	247	266	4.000	NA
2BG9	B	223	239	4.000	NA	2BG9	B	250	274	4.000	NA	2BG9	B	288	301	4.000	NA
2BG9	B	433	465	4.000	NA	2BG9	C	291	314	4.000	NA	2BG9	C	453	477	4.000	NA
2BG9	C	233	250	4.000	NA	2BG9	C	257	280	4.000	NA	2BG9	E	285	305	4.000	NA
2BG9	E	454	471	4.000	NA	2BG9	E	222	236	4.000	NA	2BG9	E	253	277	4.000	NA
2BHW	A	55	86	2.500	0.22	2BHW	A	124	143	2.500	0.22	2BHW	A	170	200	2.500	0.22
2BL2	A	12	45	2.100	0.19	2BL2	A	52	78	2.100	0.19	2BL2	A	86	122	2.100	0.19
2BL2	A	128	155	2.100	0.19	2C3E	A	8	37	2.800	0.25	2C3E	A	210	238	2.800	0.25
2C3E	A	275	290	2.800	0.25	2C3E	A	74	98	2.800	0.25	2C3E	A	108	126	2.800	0.25
2C3E	A	169	200	2.800	0.25	2E74	A	35	55	3.000	0.23	2E74	A	79	105	3.000	0.23
2E74	A	114	135	3.000	0.23	2E74	A	177	209	3.000	0.23	2E74	B	39	57	3.000	0.23
2E74	B	94	116	3.000	0.23	2E74	B	127	145	3.000	0.23	2EVU	A	4	33	2.300	0.19
2EVU	A	52	72	2.300	0.19	2EVU	A	99	122	2.300	0.19	2EVU	A	143	164	2.300	0.19
2EVU	A	176	188	2.300	0.19	2EVU	A	200	212	2.300	0.19	2EVU	A	227	243	2.300	0.19
2EZ0	A	33	63	3.540	0.26	2EZ0	A	75	97	3.540	0.26	2EZ0	A	152	165	3.540	0.26
2EZ0	A	215	231	3.540	0.26	2EZ0	A	253	282	3.540	0.26	2EZ0	A	290	305	3.540	0.26
2EZ0	A	330	348	3.540	0.26	2EZ0	A	359	378	3.540	0.26	2EZ0	A	387	399	3.540	0.26
2EZ0	A	421	434	3.540	0.26	2EZ0	A	171	189	3.540	0.26	2FBW	C	34	62	2.100	0.19
2FBW	C	81	109	2.100	0.19	2FBW	C	116	138	2.100	0.19	2FYN	A	46	66	3.200	0.22
2FYN	A	91	118	3.200	0.22	2FYN	A	126	148	3.200	0.22	2FYN	A	189	217	3.200	0.22
2FYN	A	328	345	3.200	0.22	2FYN	A	363	381	3.200	0.22	2FYN	A	390	412	3.200	0.22
2FYN	A	251	269	3.200	0.22	2FYN	B	222	251	3.200	0.22	2FYN	C	12	36	3.200	0.22
2GFP	A	10	33	3.500	0.28	2GFP	A	43	56	3.500	0.28	2GFP	A	208	226	3.500	0.28
2GFP	A	242	257	3.500	0.28	2GFP	A	270	282	3.500	0.28	2GFP	A	294	312	3.500	0.28
2GFP	A	321	343	3.500	0.28	2GFP	A	357	377	3.500	0.28	2GFP	A	100	126	3.500	0.28
2GFP	A	134	150	3.500	0.28	2GFP	A	160	175	3.500	0.28	2HYD	A	278	317	3.000	0.26
2HYD	A	12	41	3.000	0.26	2HYD	A	52	106	3.000	0.26	2HYD	A	111	159	3.000	0.26
2HYD	A	164	209	3.000	0.26	2HYD	A	218	272	3.000	0.26	2IC8	A	201	217	2.100	0.24

Helices in membrane set. Code = PDB Code; C = Chain identifier; St. = Number of first residue in helix; End = Number of last residue in helix; Res. = Resolution (Angstroms); R = R-Factor. NAs in the last two columns indicate the protein structure was solved by a method other that X-ray diffraction.

Table S5 continued. Membrane Helices

Code	C	St.	End	Res.	R	Code	C	St.	End	Res.	R	Code	C	St.	End	Res.	R
2IC8	A	229	241	2.100	0.24	2IC8	A	251	268	2.100	0.24	2IC8	A	95	112	2.100	0.24
2IC8	A	152	167	2.100	0.24	2IC8	A	171	192	2.100	0.24	2IUB	F	278	307	2.900	0.26
2IUB	F	328	342	2.900	0.26	2JAF	A	28	50	1.700	0.24	2JAF	A	58	81	1.700	0.24
2JAF	A	106	125	1.700	0.24	2JAF	A	131	153	1.700	0.24	2JAF	A	158	188	1.700	0.24
2JAF	A	193	216	1.700	0.24	2JAF	A	227	252	1.700	0.24	2K37	A	5	26	NA	NA
2K73	A	42	61	NA	NA	2K73	A	69	96	NA	NA	2K73	A	142	162	NA	NA
2K73	A	12	35	NA	NA	2K9P	A	39	72	NA	NA	2K9P	A	80	103	NA	NA
2K9Y	A	537	553	NA	NA	2KNC	B	701	739	NA	NA	2KS1	B	148	169	NA	NA
2KS9	A	29	58	NA	NA	2KS9	A	66	95	NA	NA	2KS9	A	102	135	NA	NA
2KS9	A	144	162	NA	NA	2KS9	A	193	220	NA	NA	2KS9	A	239	273	NA	NA
2KS9	A	285	307	NA	NA	2KSD	A	28	45	NA	NA	2KSE	A	15	33	NA	NA
2KSE	A	160	180	NA	NA	2KSY	A	5	27	NA	NA	2KSY	A	34	54	NA	NA
2KSY	A	123	148	NA	NA	2KSY	A	155	179	NA	NA	2KSY	A	70	91	NA	NA
2KSY	A	95	114	NA	NA	2L0J	A	25	44	NA	NA	2L2T	A	51	76	NA	NA
2L35	A	9	29	NA	NA	2L35	A	39	55	NA	NA	2L6X	A	27	51	NA	NA
2L6X	A	60	85	NA	NA	2L6X	A	91	113	NA	NA	2L6X	A	122	139	NA	NA
2L6X	A	180	204	NA	NA	2L9U	A	641	671	NA	NA	2LCK	A	16	40	NA	NA
2LCK	A	214	237	NA	NA	2LCK	A	267	295	NA	NA	2LCK	A	83	107	NA	NA
2LCK	A	116	131	NA	NA	2LCK	A	172	196	NA	NA	2LKG	A	15	41	NA	NA
2LKG	A	52	69	NA	NA	2LKG	A	79	97	NA	NA	2LLY	A	8	30	NA	NA
2LLY	A	37	58	NA	NA	2LLY	A	71	92	NA	NA	2LLY	A	110	129	NA	NA
2LNL	A	38	66	NA	NA	2LNL	A	75	98	NA	NA	2LNL	A	112	136	NA	NA
2LNL	A	150	173	NA	NA	2LNL	A	207	225	NA	NA	2LNL	A	239	267	NA	NA
2LNL	A	279	307	NA	NA	2LOM	A	30	46	NA	NA	2LOM	A	65	81	NA	NA
2LOR	A	65	95	NA	NA	2LOR	A	19	52	NA	NA	2NQ2	A	7	24	2.400	0.22
2NQ2	A	53	86	2.400	0.22	2NQ2	A	99	112	2.400	0.22	2NQ2	A	235	256	2.400	0.22
2NQ2	A	311	327	2.400	0.22	2NQ2	A	117	139	2.400	0.22	2NQ2	A	147	170	2.400	0.22
2NQ2	A	194	214	2.400	0.22	2NQ2	A	282	303	2.400	0.22	2NR9	A	116	131	2.200	0.24
2NR9	A	166	191	2.200	0.24	2NR9	A	10	26	2.200	0.24	2NR9	A	63	82	2.200	0.24
2NR9	A	86	108	2.200	0.24	2OAR	A	69	99	3.500	0.32	2OAR	A	13	44	3.500	0.32
2ONK	C	2	29	3.100	0.26	2ONK	C	35	77	3.100	0.26	2ONK	C	169	198	3.100	0.26
2ONK	C	130	152	3.100	0.26	2ONK	C	226	249	3.100	0.26	2Q6H	A	166	183	1.850	0.20
2Q6H	A	191	214	1.850	0.20	2Q6H	A	241	255	1.850	0.20	2Q6H	A	276	290	1.850	0.20
2Q6H	A	337	368	1.850	0.20	2Q6H	A	449	477	1.850	0.20	2Q6H	A	483	509	1.850	0.20
2Q6H	A	11	36	1.850	0.20	2Q6H	A	44	71	1.850	0.20	2Q6H	A	88	123	1.850	0.20
2Q6H	A	375	390	1.850	0.20	2Q6H	A	399	423	1.850	0.20	2Q7M	C	3	35	4.250	0.24
2Q7M	C	117	138	4.250	0.24	2Q7M	C	50	73	4.250	0.24	2Q7M	C	81	100	4.250	0.24
2QDZ	A	10	26	3.150	0.29	2QFI	A	129	141	3.800	0.32	2QKS	A	103	132	2.200	0.23
2QKS	A	42	65	2.200	0.23	2QTS	E	57	69	1.900	0.21	2QTS	E	427	460	1.900	0.21
2R6G	F	69	90	2.800	0.24	2R6G	F	277	306	2.800	0.24	2R6G	F	484	503	2.800	0.24
2R6G	F	18	35	2.800	0.24	2R6G	F	40	55	2.800	0.24	2R6G	F	365	388	2.800	0.24
2R6G	F	426	447	2.800	0.24	2R9R	H	221	243	2.400	0.21	2R9R	H	254	274	2.400	0.21
2R9R	H	381	416	2.400	0.21	2R9R	H	160	183	2.400	0.21	2R9R	H	279	296	2.400	0.21
2R9R	H	321	346	2.400	0.21	2RH1	A	103	136	2.400	0.20	2RH1	A	147	169	2.400	0.20
2RH1	A	197	229	2.400	0.20	2RH1	A	267	298	2.400	0.20	2RH1	A	31	59	2.400	0.20
2RH1	A	67	95	2.400	0.20	2RH1	A	305	326	2.400	0.20	2UUI	A	5	32	2.000	0.20
2UUI	A	105	141	2.000	0.20	2UUI	A	44	72	2.000	0.20	2UUI	A	76	98	2.000	0.20
2VV5	A	29	58	3.450	0.29	2VV5	A	63	84	3.450	0.29	2VV5	A	111	125	3.450	0.29
2W1P	A	41	69	1.400	0.16	2W1P	A	82	103	1.400	0.16	2W1P	A	127	151	1.400	0.16
2W1P	A	168	188	1.400	0.16	2W1P	A	200	215	1.400	0.16	2W1P	A	247	265	1.400	0.16
2WIE	A	5	41	2.130	0.20	2WIE	A	46	76	2.130	0.20	2WLL	B	121	149	3.650	0.25
2WLL	B	60	83	3.650	0.25	2WPD	J	7	36	3.430	0.29	2WPD	J	47	73	3.430	0.29
2WSC	4	42	55	3.300	0.36	2WSW	A	128	161	2.290	0.21	2WSW	A	312	337	2.290	0.21
2WSW	A	344	375	2.290	0.21	2WSW	A	404	434	2.290	0.21	2WSW	A	16	30	2.290	0.21
2WSW	A	103	117	2.290	0.21	2WSW	A	187	222	2.290	0.21	2WSW	A	229	247	2.290	0.21
2WSW	A	446	465	2.290	0.21	2WSW	A	469	499	2.290	0.21	2WSW	A	33	48	2.290	0.21
2WSW	A	52	71	2.290	0.21	2WSW	A	252	275	2.290	0.21	2WSW	A	279	292	2.290	0.21
2X2V	A	3	33	2.500	0.19	2X2V	A	38	68	2.500	0.19	2X79	A	60	86	3.800	0.28
2X79	A	105	133	3.800	0.28	2X79	A	210	231	3.800	0.28	2X79	A	244	273	3.800	0.28
2X79	A	307	328	3.800	0.28	2X79	A	336	356	3.800	0.28	2X79	A	428	448	3.800	0.28
2X79	A	143	157	3.800	0.28	2X79	A	163	189	3.800	0.28	2XND	J	18	34	3.500	0.26
2XND	J	51	70	3.500	0.26	2XQ2	A	11	28	2.730	0.25	2XQ2	A	280	313	2.730	0.25
2XQ2	A	453	472	2.730	0.25	2XQ2	A	479	501	2.730	0.25	2XQ2	A	522	544	2.730	0.25
2XQ2	A	549	572	2.730	0.25	2XQ2	A	85	108	2.730	0.25	2XQ2	A	163	177	2.730	0.25
2XQ2	A	187	209	2.730	0.25	2XQ2	A	349	378	2.730	0.25	2XQ2	A	392	413	2.730	0.25
2XQ2	A	423	447	2.730	0.25	2XQ2	A	124	156	2.730	0.25	2YEV	A	64	99	2.360	0.17
2YEV	A	107	125	2.360	0.17	2YEV	A	153	179	2.360	0.17	2YEV	A	193	224	2.360	0.17
2YEV	A	239	272	2.360	0.17	2YEV	A	279	292	2.360	0.17	2YEV	A	308	336	2.360	0.17
2YEV	A	416	442	2.360	0.17	2YEV	A	459	489	2.360	0.17	2YEV	A	558	573	2.360	0.17
2YEV	A	580	597	2.360	0.17	2YEV	A	617	643	2.360	0.17	2YEV	A	724	756	2.360	0.17

Helices in membrane set. Code = PDB Code; C = Chain identifier; St. = Number of first residue in helix; End = Number of last residue in helix; Res. = Resolution (Angstroms); R = R-Factor. NAs in the last two columns indicate the protein structure was solved by a method other that X-ray diffraction.

Table S5 continued. Membrane Helices

Code	C	St.	End	Res.	R	Code	C	St.	End	Res.	R	Code	C	St.	End	Res.	R
2YEV	A	765	787	2.360	0.17	2YEV	A	25	52	2.360	0.17	2YEV	A	345	368	2.360	0.17
2YEV	A	381	409	2.360	0.17	2YEV	A	657	681	2.360	0.17	2YEV	A	686	715	2.360	0.17
2YEV	B	30	62	2.360	0.17	2YEV	B	81	109	2.360	0.17	2YIU	B	248	277	2.700	0.24
2YIU	C	19	39	2.700	0.24	2YN6	A	261	285	3.310	0.23	2YN6	A	202	220	3.310	0.23
2YN6	A	228	252	3.310	0.23	2YVX	A	278	303	3.500	0.29	2YVX	A	323	343	3.500	0.29
2YVX	A	352	374	3.500	0.29	2YVX	A	389	414	3.500	0.29	2YVX	A	429	443	3.500	0.29
2ZJS	Y	151	177	3.200	0.25	2ZJS	Y	183	205	3.200	0.25	2ZJS	Y	215	236	3.200	0.25
2ZJS	Y	272	288	3.200	0.25	2ZJS	Y	309	327	3.200	0.25	2ZJS	Y	356	388	3.200	0.25
2ZJS	Y	401	417	3.200	0.25	2ZJS	Y	14	31	3.200	0.25	2ZJS	Y	105	132	3.200	0.25
2ZJS	Y	78	92	3.200	0.25	2ZT9	C	252	283	3.000	0.23	2ZW3	A	23	42	3.500	0.34
2ZW3	A	73	105	3.500	0.34	2ZW3	A	132	156	3.500	0.34	2ZW3	A	185	215	3.500	0.34
2ZXE	G	22	38	2.400	0.25	3AM6	A	77	92	3.200	0.29	3AM6	A	101	122	3.200	0.29
3AM6	A	129	150	3.200	0.29	3AM6	A	161	187	3.200	0.29	3AM6	A	196	226	3.200	0.29
3AM6	A	7	30	3.200	0.29	3AM6	A	39	61	3.200	0.29	3AON	A	31	69	2.000	0.20
3AON	A	127	162	2.000	0.20	3AQP	A	4	23	3.300	0.30	3AQP	A	326	353	3.300	0.30
3AQP	A	364	390	3.300	0.30	3AQP	A	395	428	3.300	0.30	3AQP	A	449	468	3.300	0.30
3AQP	A	621	650	3.300	0.30	3AQP	A	265	292	3.300	0.30	3AQP	A	296	319	3.300	0.30
3AQP	A	565	586	3.300	0.30	3AQP	A	594	615	3.300	0.30	3AQP	A	658	685	3.300	0.30
3AQP	A	692	725	3.300	0.30	3ARC	j	10	31	1.900	0.17	3AYF	A	20	46	2.500	0.24
3AYF	A	248	274	2.500	0.24	3AYF	A	300	329	2.500	0.24	3AYF	A	349	379	2.500	0.24
3AYF	A	386	413	2.500	0.24	3AYF	A	435	462	2.500	0.24	3AYF	A	469	482	2.500	0.24
3AYF	A	496	529	2.500	0.24	3AYF	A	535	548	2.500	0.24	3AYF	A	572	601	2.500	0.24
3AYF	A	609	639	2.500	0.24	3AYF	A	647	673	2.500	0.24	3AYF	A	683	716	2.500	0.24
3AYF	A	735	763	2.500	0.24	3B4R	A	19	31	3.300	0.25	3B4R	A	40	63	3.300	0.25
3B4R	A	96	112	3.300	0.25	3B4R	A	125	138	3.300	0.25	3B4R	A	163	185	3.300	0.25
3B4R	A	192	217	3.300	0.25	3B8C	A	242	256	3.600	0.35	3B8C	A	630	650	3.600	0.35
3B8E	A	123	137	3.500	0.28	3B8E	A	276	289	3.500	0.28	3B8E	A	310	322	3.500	0.28
3B8E	A	755	787	3.500	0.28	3B8E	A	851	866	3.500	0.28	3B8E	A	913	928	3.500	0.28
3B8E	A	945	963	3.500	0.28	3B8E	A	987	1003	3.500	0.28	3B9Y	A	34	59	1.850	0.16
3B9Y	A	66	89	1.850	0.16	3B9Y	A	102	120	1.850	0.16	3B9Y	A	130	148	1.850	0.16
3B9Y	A	198	220	1.850	0.16	3B9Y	A	227	253	1.850	0.16	3B9Y	A	260	274	1.850	0.16
3B9Y	A	283	310	1.850	0.16	3B9Y	A	325	338	1.850	0.16	3B9Y	A	343	369	1.850	0.16
3B9Y	A	170	182	1.850	0.16	3C02	A	9	35	2.050	0.18	3C02	A	43	61	2.050	0.18
3C02	A	134	156	2.050	0.18	3C02	A	166	183	2.050	0.18	3C02	A	87	107	2.050	0.18
3C02	A	221	248	2.050	0.18	3C1G	A	97	119	2.300	0.19	3C1G	A	125	147	2.300	0.19
3C1G	A	168	180	2.300	0.19	3C1G	A	199	219	2.300	0.19	3C1G	A	225	252	2.300	0.19
3C1G	A	258	272	2.300	0.19	3C1G	A	348	380	2.300	0.19	3C1G	A	7	32	2.300	0.19
3C1G	A	282	300	2.300	0.19	3C1G	A	313	330	2.300	0.19	3C1G	A	40	58	2.300	0.19
3CHX	A	239	259	3.900	0.34	3CHX	B	24	49	3.900	0.34	3CHX	B	146	166	3.900	0.34
3CHX	C	69	85	3.900	0.34	3CHX	C	100	122	3.900	0.34	3CHX	C	144	163	3.900	0.34
3CWB	E	32	58	3.510	0.28	3CWB	G	33	68	3.510	0.28	3CX5	D	263	296	1.900	0.24
3CX5	I	18	43	1.900	0.24	3D31	C	242	255	3.000	0.25	3D31	C	14	41	3.000	0.25
3D31	C	140	165	3.000	0.25	3D31	C	193	216	3.000	0.25	3D31	C	56	86	3.000	0.25
3D9S	A	8	32	2.000	0.16	3D9S	A	84	108	2.000	0.16	3D9S	A	128	150	2.000	0.16
3D9S	A	161	174	2.000	0.16	3D9S	A	204	221	2.000	0.16	3D9S	A	42	56	2.000	0.16
3DDL	A	91	110	1.900	0.25	3DDL	A	119	141	1.900	0.25	3DDL	A	151	171	1.900	0.25
3DDL	A	183	208	1.900	0.25	3DDL	A	222	257	1.900	0.25	3DDL	A	10	38	1.900	0.25
3DDL	A	46	72	1.900	0.25	3DIN	C	82	95	4.500	0.28	3DIN	C	111	139	4.500	0.28
3DIN	C	159	178	4.500	0.28	3DIN	C	186	198	4.500	0.28	3DIN	C	214	228	4.500	0.28
3DIN	C	274	286	4.500	0.28	3DIN	C	304	321	4.500	0.28	3DIN	C	350	380	4.500	0.28
3DIN	C	392	412	4.500	0.28	3DIN	D	32	57	4.500	0.28	3DIN	E	15	30	4.500	0.28
3DIN	E	55	70	4.500	0.28	3EAM	A	201	213	2.900	0.20	3EAM	A	221	244	2.900	0.20
3EAM	A	254	281	2.900	0.20	3EAM	A	286	314	2.900	0.20	3EGW	C	3	30	1.900	0.17
3EGW	C	50	68	1.900	0.17	3EGW	C	182	197	1.900	0.17	3EGW	C	154	167	1.900	0.17
3EGW	C	124	147	1.900	0.17	3EGW	C	83	111	1.900	0.17	3EML	A	74	106	2.600	0.20
3EML	A	118	138	2.600	0.20	3EML	A	174	204	2.600	0.20	3EML	A	222	258	2.600	0.20
3EML	A	7	33	2.600	0.20	3EML	A	43	66	2.600	0.20	3EML	A	267	290	2.600	0.20
3EMO	A	1019	1035	3.000	0.22	3GI9	C	10	25	2.480	0.25	3GI9	C	122	138	2.480	0.25
3GI9	C	144	166	2.480	0.25	3GI9	C	40	62	2.480	0.25	3GI9	C	85	112	2.480	0.25
3GI9	C	184	211	2.480	0.25	3GI9	C	221	244	2.480	0.25	3GI9	C	271	304	2.480	0.25
3GI9	C	322	336	2.480	0.25	3GI9	C	340	365	2.480	0.25	3GI9	C	373	395	2.480	0.25
3GI9	C	399	422	2.480	0.25	3HD6	A	10	29	2.100	0.17	3HD6	A	59	77	2.100	0.17
3HD6	A	85	109	2.100	0.17	3HD6	A	122	140	2.100	0.17	3HD6	A	148	169	2.100	0.17
3HD6	A	215	231	2.100	0.17	3HD6	A	243	270	2.100	0.17	3HD6	A	280	292	2.100	0.17
3HD6	A	303	330	2.100	0.17	3HD6	A	345	359	2.100	0.17	3HD6	A	385	416	2.100	0.17
3HD7	F	226	272	3.400	0.24	3HZQ	A	15	45	3.820	0.29	3HZQ	A	65	87	3.820	0.29
3KBC	A	13	29	3.510	0.27	3KBC	A	37	71	3.510	0.27	3KBC	A	83	106	3.510	0.27
3KBC	A	151	169	3.510	0.27	3KBC	A	175	218	3.510	0.27	3KBC	A	225	251	3.510	0.27
3KBC	A	261	275	3.510	0.27	3KBC	A	314	329	3.510	0.27	3KBC	A	377	414	3.510	0.27
3KLY	C	28	54	2.100	0.18	3KLY	C	63	83	2.100	0.18	3KLY	C	107	134	2.100	0.18

Helices in membrane set. Code = PDB Code; C = Chain identifier; St. = Number of first residue in helix; End = Number of last residue in helix; Res. = Resolution (Angstroms); R = R-Factor. NAs in the last two columns indicate the protein structure was solved by a method other that X-ray diffraction.

Table S5 continued. Membrane Helices

Code	C	St.	End	Res.	R	Code	C	St.	End	Res.	R	Code	C	St.	End	Res.	R
3KLY	C	160	181	2.100	0.18	3KLY	C	187	202	2.100	0.18	3KLY	C	246	277	2.100	0.18
3KLY	C	209	224	2.100	0.18	3KP9	A	21	43	3.600	0.26	3KP9	A	72	88	3.600	0.26
3KP9	A	103	123	3.600	0.26	3KP9	A	131	147	3.600	0.26	3KP9	A	157	179	3.600	0.26
3KVN	A	314	337	2.500	0.21	3KZI	F	19	40	3.600	0.30	3M6E	A	10	24	2.650	0.20
3M6E	A	99	120	2.650	0.20	3M6E	A	261	286	2.650	0.20	3M6E	A	295	308	2.650	0.20
3M6E	A	209	228	2.650	0.20	3M6E	A	46	64	2.650	0.20	3MC9	B	335	351	2.200	0.20
3MP7	A	30	45	2.900	0.28	3MP7	A	116	130	2.900	0.28	3MP7	A	147	168	2.900	0.28
3MP7	A	176	193	2.900	0.28	3MP7	A	241	259	2.900	0.28	3MP7	A	287	307	2.900	0.28
3MP7	A	345	368	2.900	0.28	3MP7	A	410	427	2.900	0.28	3MP7	A	434	455	2.900	0.28
3MP7	B	29	58	2.900	0.28	3NE2	C	5	34	3.000	0.25	3NE2	C	53	73	3.000	0.25
3NE2	C	100	123	3.000	0.25	3NE2	C	146	164	3.000	0.25	3NE2	C	176	189	3.000	0.25
3NE2	C	201	213	3.000	0.25	3NE2	C	222	243	3.000	0.25	3O7Q	A	25	48	3.140	0.22
3O7Q	A	151	171	3.140	0.22	3O7Q	A	196	228	3.140	0.22	3O7Q	A	259	287	3.140	0.22
3O7Q	A	117	144	3.140	0.22	3O7Q	A	62	86	3.140	0.22	3O7Q	A	90	112	3.140	0.22
3O7Q	A	294	319	3.140	0.22	3O7Q	A	324	343	3.140	0.22	3O7Q	A	349	373	3.140	0.22
3O7Q	A	380	402	3.140	0.22	3O7Q	A	415	429	3.140	0.22	3OB6	B	42	66	3.000	0.24
3OB6	B	275	306	3.000	0.24	3OB6	B	324	339	3.000	0.24	3OB6	B	83	111	3.000	0.24
3OB6	B	122	141	3.000	0.24	3OB6	B	225	246	3.000	0.24	3OB6	B	352	374	3.000	0.24
3OB6	B	146	159	3.000	0.24	3OB6	B	383	403	3.000	0.24	3OB6	B	407	426	3.000	0.24
3ORG	A	278	292	3.500	0.26	3ORG	A	321	350	3.500	0.26	3ORG	A	359	371	3.500	0.26
3ORG	A	430	451	3.500	0.26	3ORG	A	460	473	3.500	0.26	3ORG	A	90	129	3.500	0.26
3ORG	A	135	158	3.500	0.26	3ORG	A	210	225	3.500	0.26	3ORG	A	237	255	3.500	0.26
3P5N	A	11	27	3.600	0.27	3P5N	A	60	77	3.600	0.27	3P5N	A	84	100	3.600	0.27
3P5N	A	110	130	3.600	0.27	3P5N	A	154	182	3.600	0.27	3PBL	A	35	56	2.890	0.24
3PBL	A	63	91	2.890	0.24	3PBL	A	148	166	2.890	0.24	3PBL	A	186	215	2.890	0.24
3PBL	A	322	352	2.890	0.24	3PBL	A	100	132	2.890	0.24	3PBL	A	362	385	2.890	0.24
3PIK	A	63	79	2.300	0.20	3PIK	A	122	138	2.300	0.20	3PIK	A	269	285	2.300	0.20
3PIK	A	330	346	2.300	0.20	3PL9	A	90	116	2.800	0.28	3PL9	A	145	163	2.800	0.28
3PL9	A	190	215	2.800	0.28	3PUX	G	11	39	2.300	0.23	3PUX	G	81	110	2.300	0.23
3PUX	G	260	281	2.300	0.23	3PUX	G	212	227	2.300	0.23	3Q7K	J	33	56	2.800	0.22
3Q7K	J	65	83	2.800	0.22	3Q7K	J	107	135	2.800	0.22	3Q7K	J	161	182	2.800	0.22
3Q7K	J	188	203	2.800	0.22	3Q7K	J	247	265	2.800	0.22	3Q7K	J	210	225	2.800	0.22
3QBG	A	62	86	1.800	0.23	3QBG	A	122	141	1.800	0.23	3QBG	A	146	168	1.800	0.23
3QBG	A	241	270	1.800	0.23	3QBG	A	32	55	1.800	0.23	3QBG	A	174	200	1.800	0.23
3QBG	A	208	231	1.800	0.23	3QF4	A	270	311	2.900	0.22	3QF4	A	13	41	2.900	0.22
3QF4	A	51	98	2.900	0.22	3QF4	A	210	263	2.900	0.22	3QF4	A	109	151	2.900	0.22
3QF4	A	158	197	2.900	0.22	3QF4	B	308	333	2.900	0.22	3QF4	B	35	66	2.900	0.22
3QF4	B	75	120	2.900	0.22	3QF4	B	236	285	2.900	0.22	3QF4	B	133	174	2.900	0.22
3QF4	B	177	221	2.900	0.22	3RGB	C	51	72	2.800	0.27	3RGB	C	89	113	2.800	0.27
3RGB	C	127	162	2.800	0.27	3RGB	C	171	197	2.800	0.27	3RGB	C	257	269	2.800	0.27
3RIF	A	214	230	3.340	0.25	3RIF	A	242	264	3.340	0.25	3RIF	A	275	301	3.340	0.25
3RIF	A	308	338	3.340	0.25	3RKO	A	17	42	3.000	0.23	3RKO	A	64	89	3.000	0.23
3RKO	A	97	119	3.000	0.23	3RLB	B	7	25	2.000	0.21	3RLB	B	78	91	2.000	0.21
3RLB	B	107	131	2.000	0.21	3RLB	B	143	172	2.000	0.21	3RLB	B	53	68	2.000	0.21
3RW0	A	1116	1151	2.950	0.27	3RW0	A	1198	1216	2.950	0.27	3RW0	A	1001	1032	2.950	0.27
3RW0	A	1042	1065	2.950	0.27	3RZE	A	33	54	3.100	0.22	3RZE	A	63	89	3.100	0.22
3RZE	A	98	130	3.100	0.22	3RZE	A	143	162	3.100	0.22	3RZE	A	188	216	3.100	0.22
3RZE	A	450	467	3.100	0.22	3RZE	A	408	437	3.100	0.22	3RZE	A	1126	1140	3.100	0.22
3SFE	C	37	65	2.810	0.27	3SFE	C	84	113	2.810	0.27	3SFE	C	120	141	2.810	0.27
3SPG	A	156	185	2.610	0.20	3SPG	A	79	108	2.610	0.20	3SYA	A	169	196	2.980	0.24
3SYA	A	91	119	2.980	0.24	3TDO	C	60	78	2.200	0.18	3TDO	C	83	95	2.200	0.18
3TDO	C	101	128	2.200	0.18	3TDO	C	180	195	2.200	0.18	3TDO	C	202	214	2.200	0.18
3TDO	C	224	253	2.200	0.18	3TDO	C	2	20	2.200	0.18	3TDO	C	24	55	2.200	0.18
3TDO	C	136	149	2.200	0.18	3TDO	C	153	175	2.200	0.18	3TUF	A	105	126	2.260	0.17
3TUF	A	139	164	2.260	0.17	3TUI	A	3	40	2.900	0.24	3TUI	A	94	112	2.900	0.24
3TUI	A	186	211	2.900	0.24	3TUI	A	51	63	2.900	0.24	3TUI	A	68	82	2.900	0.24
3TUI	A	144	163	2.900	0.24	3UDC	A	17	49	3.350	0.25	3UDC	A	60	87	3.350	0.25
3UDC	A	92	125	3.350	0.25	3UG9	A	158	174	2.300	0.21	3UG9	A	246	271	2.300	0.21
3UG9	A	281	313	2.300	0.21	3UG9	A	86	107	2.300	0.21	3UG9	A	120	138	2.300	0.21
3UG9	A	186	206	2.300	0.21	3UG9	A	211	241	2.300	0.21	3UON	A	93	126	3.000	0.23
3UON	A	137	166	3.000	0.23	3UON	A	184	213	3.000	0.23	3UON	A	384	411	3.000	0.23
3UON	A	23	49	3.000	0.23	3UON	A	57	85	3.000	0.23	3UON	A	419	442	3.000	0.23
3UX4	A	3	21	3.260	0.24	3UX4	A	26	51	3.260	0.24	3UX4	A	142	159	3.260	0.24
3UX4	A	170	187	3.260	0.24	3UX4	A	76	95	3.260	0.24	3UX4	A	101	122	3.260	0.24
3V2Y	A	42	72	2.800	0.23	3V2Y	A	79	104	2.800	0.23	3V2Y	A	114	145	2.800	0.23
3V2Y	A	158	174	2.800	0.23	3V2Y	A	200	231	2.800	0.23	3V2Y	A	252	280	2.800	0.23
3V2Y	A	294	311	2.800	0.23	3V6I	B	73	102	2.250	0.23	3VOU	A	74	98	3.200	0.29
3VOU	A	22	44	3.200	0.29	3VR8	C	71	94	2.810	0.23	3VR8	C	116	142	2.810	0.23
3VR8	C	149	179	2.810	0.23	3VR8	D	116	141	2.810	0.23	3VR8	D	56	76	2.810	0.23
3VR8	D	82	106	2.810	0.23	3VW7	A	172	205	2.200	0.22	3VW7	A	216	235	2.200	0.22

Helices in membrane set. Code = PDB Code; C = Chain identifier; St. = Number of first residue in helix; End = Number of last residue in helix; Res. = Resolution (Angstroms); R = R-Factor. NAs in the last two columns indicate the protein structure was solved by a method other that X-ray diffraction.

Table S5 continued. Membrane Helices

Code	C	St.	End	Res.	R	Code	C	St.	End	Res.	R	Code	C	St.	End	Res.	R
3VW7	A	266	296	2.200	0.22	3VW7	A	305	338	2.200	0.22	3VW7	A	347	373	2.200	0.22
3VW7	A	92	131	2.200	0.22	3VW7	A	136	163	2.200	0.22	3ZUX	A	251	276	2.200	0.20
3ZUX	A	284	308	2.200	0.20	3ZUX	A	15	27	2.200	0.20	3ZUX	A	36	54	2.200	0.20
3ZUX	A	67	91	2.200	0.20	3ZUX	A	125	137	2.200	0.20	3ZUX	A	155	183	2.200	0.20
3ZUX	A	188	214	2.200	0.20	3ZUX	A	217	245	2.200	0.20	4A2N	B	4	28	3.400	0.24
4A2N	B	41	61	3.400	0.24	4A2N	B	75	99	3.400	0.24	4A2N	B	127	142	3.400	0.24
4A2N	B	150	172	3.400	0.24	4AMJ	B	205	236	2.300	0.20	4AMJ	B	278	313	2.300	0.20
4AMJ	B	33	67	2.300	0.20	4AMJ	B	78	104	2.300	0.20	4AMJ	B	111	142	2.300	0.20
4AMJ	B	322	343	2.300	0.20	4AMJ	B	156	178	2.300	0.20	4APS	A	15	46	3.300	0.27
4APS	A	110	136	3.300	0.27	4APS	A	216	241	3.300	0.27	4APS	A	248	266	3.300	0.27
4APS	A	285	310	3.300	0.27	4APS	A	329	342	3.300	0.27	4APS	A	355	376	3.300	0.27
4APS	A	388	401	3.300	0.27	4APS	A	425	439	3.300	0.27	4APS	A	452	470	3.300	0.27
4APS	A	53	75	3.300	0.27	4APS	A	84	103	3.300	0.27	4APS	A	145	171	3.300	0.27
4APS	A	175	198	3.300	0.27	4AYX	A	428	469	2.900	0.23	4AYX	A	167	201	2.900	0.23
4AYX	A	208	257	2.900	0.23	4AYX	A	268	309	2.900	0.23	4AYX	A	369	422	2.900	0.23
4AYX	A	312	357	2.900	0.23	4B4A	A	60	88	3.500	0.25	4B4A	A	101	128	3.500	0.25
4B4A	A	146	175	3.500	0.25	4B4A	A	6	27	3.500	0.25	4B4A	A	31	44	3.500	0.25
4B4A	A	181	202	3.500	0.25	4B4A	A	207	225	3.500	0.25	4DAJ	D	138	170	3.400	0.25
4DAJ	D	182	210	3.400	0.25	4DAJ	D	229	254	3.400	0.25	4DAJ	D	494	513	3.400	0.25
4DAJ	D	66	94	3.400	0.25	4DAJ	D	101	129	3.400	0.25	4DAJ	D	522	544	3.400	0.25
4DJH	B	219	256	2.900	0.23	4DJH	B	271	299	2.900	0.23	4DJH	B	57	86	2.900	0.23
4DJH	B	93	121	2.900	0.23	4DJH	B	129	160	2.900	0.23	4DJH	B	309	332	2.900	0.23
4DJH	B	173	196	2.900	0.23	4DJI	A	41	65	3.190	0.31	4DJI	A	103	117	3.190	0.31
4DJI	A	127	146	3.190	0.31	4DJI	A	154	180	3.190	0.31	4DJI	A	235	259	3.190	0.31
4DJI	A	364	393	3.190	0.31	4DJI	A	410	429	3.190	0.31	4DJI	A	289	321	3.190	0.31
4DL0	G	9	58	2.910	0.21	4DVE	A	3	23	2.090	0.19	4DVE	A	55	70	2.090	0.19
4DVE	A	149	179	2.090	0.19	4DVE	A	90	109	2.090	0.19	4DVE	A	118	143	2.090	0.19
4DXW	B	12	27	3.050	0.24	4DXW	B	38	64	3.050	0.24	4DXW	B	196	224	3.050	0.24
4DXW	B	117	154	3.050	0.24	4EA3	B	49	76	3.010	0.25	4EA3	B	85	113	3.010	0.25
4EA3	B	120	153	3.010	0.25	4EA3	B	164	188	3.010	0.25	4EA3	B	214	241	3.010	0.25
4EA3	B	259	287	3.010	0.25	4EA3	B	295	320	3.010	0.25	4EIY	A	174	208	1.800	0.18
4EIY	A	220	257	1.800	0.18	4EIY	A	4	33	1.800	0.18	4EIY	A	43	67	1.800	0.18
4EIY	A	74	106	1.800	0.18	4EIY	A	111	138	1.800	0.18	4EIY	A	267	290	1.800	0.18
4EIY	A	293	305	1.800	0.18	4EJ4	A	45	75	3.400	0.26	4EJ4	A	83	110	3.400	0.26
4EJ4	A	207	238	3.400	0.26	4EJ4	A	258	286	3.400	0.26	4EJ4	A	119	150	3.400	0.26
4EJ4	A	162	186	3.400	0.26	4EJ4	A	295	318	3.400	0.26	4EZC	B	49	63	2.360	0.20
4EZC	B	142	161	2.360	0.20	4EZC	B	173	186	2.360	0.20	4EZC	B	214	227	2.360	0.20
4EZC	B	301	326	2.360	0.20	4EZC	B	85	104	2.360	0.20	4EZC	B	249	268	2.360	0.20
4F4L	C	7	27	3.490	0.27	4F4L	C	74	92	3.490	0.27	4FC4	B	80	92	2.400	0.20
4FC4	B	98	125	2.400	0.20	4FC4	B	177	190	2.400	0.20	4FC4	B	219	248	2.400	0.20
4FC4	B	56	73	2.400	0.20	4FC4	B	150	172	2.400	0.20	4FC4	B	3	20	2.400	0.20
4FC4	B	23	49	2.400	0.20	4G1U	A	7	26	3.010	0.27	4G1U	A	55	79	3.010	0.27
4G1U	A	232	253	3.010	0.27	4G1U	A	279	298	3.010	0.27	4G1U	A	309	325	3.010	0.27
4G1U	A	92	104	3.010	0.27	4G1U	A	115	139	3.010	0.27	4G1U	A	145	168	3.010	0.27
4G1U	A	191	218	3.010	0.27	4GBY	A	125	152	2.810	0.23	4GBY	A	160	186	2.810	0.23
4GBY	A	191	217	2.810	0.23	4GBY	A	277	306	2.810	0.23	4GBY	A	370	384	2.810	0.23
4GBY	A	313	334	2.810	0.23	4GBY	A	341	363	2.810	0.23	4GBY	A	443	461	2.810	0.23
4GBY	A	8	29	2.810	0.23	4GBY	A	50	80	2.810	0.23	4GBY	A	84	103	2.810	0.23
4GBY	A	405	423	2.810	0.23	4GRV	A	61	87	2.800	0.23	4GRV	A	99	126	2.800	0.23
4GRV	A	139	171	2.800	0.23	4GRV	A	187	207	2.800	0.23	4GRV	A	233	265	2.800	0.23
4GRV	A	302	331	2.800	0.23	4GRV	A	341	373	2.800	0.23	4H33	A	15	37	3.100	0.28
4H33	A	68	92	3.100	0.28	4HKR	A	237	270	3.350	0.28	4HKR	A	276	323	3.350	0.28

Helices in membrane set. Code = PDB Code; C = Chain identifier; St. = Number of first residue in helix; End = Number of last residue in helix; Res. = Resolution (Angstroms); R = R-Factor. NAs in the last two columns indicate the protein structure was solved by a method other that X-ray diffraction.
